# Controller synthesis and clinical exploration of wearable gyroscopic actuators to support human balance

**DOI:** 10.1038/s41598-020-66760-w

**Published:** 2020-06-26

**Authors:** Daniel Lemus, Andrew Berry, Saher Jabeen, Chandrasekaran Jayaraman, Kristen Hohl, Frans C. T. van der Helm, Arun Jayaraman, Heike Vallery

**Affiliations:** 1 Department of Biomechanical Engineering, Delft University of Technology, Delft, 2628 CD The Netherlands; 2 Max Näder Center for Rehabilitation Technologies & Outcomes Research, Shirley Ryan AbilityLab, Chicago, IL 60611 USA

**Keywords:** Rehabilitation, Mechanical engineering

## Abstract

Gyroscopic actuators are appealing for wearable applications due to their ability to provide overground balance support without obstructing the legs. Multiple wearable robots using this actuation principle have been proposed, but none has yet been evaluated with humans. Here we use the GyBAR, a backpack-like prototype portable robot, to investigate the hypothesis that the balance of both healthy and chronic stroke subjects can be augmented through moments applied to the upper body. We quantified balance performance in terms of each participant’s ability to walk or remain standing on a narrow support surface oriented to challenge stability in either the frontal or the sagittal plane. By comparing candidate balance controllers, it was found that effective assistance did not require regulation to a reference posture. A rotational viscous field increased the distance healthy participants could walk along a 30mm-wide beam by a factor of 2.0, compared to when the GyBAR was worn but inactive. The same controller enabled individuals with chronic stroke to remain standing for a factor of 2.5 longer on a narrow block. Due to its wearability and versatility of control, the GyBAR could enable new therapy interventions for training and rehabilitation.

## Introduction

Falling is among the most frequent causes of hospitalization and death among the elderly^1^. More than 1 out of 3 adults over the age of 70 fall in a 12-month period^2^. Compared to their healthy counterparts, individuals post stroke have a sevenfold higher risk of falling^3,4^.

While it is known that impaired balance is a key risk factor for falls^5^, balance training programs for survivors of stroke have not yet been proven an effective means to actually reduce fall risk^6^. This may be due to the fact that there are many possible contributing factors to falls, including sensory deficits such as loss of proprioception, motor deficits such as paresis, or visuo-perceptual and cognitive deficits such as hemineglect^4,7^.

When provided with *external* forces from overhead support^8^, many otherwise non-ambulatory individuals can walk. This often occurs even with very low amounts of assistance, effectively aiding balance and instilling confidence. However, existing devices that could provide such support in a controlled way are bound to treadmills^9–11^, mounted to the ceiling^12–14^, or require large wheeled frames around the user, like the Andago (Hocoma AG, CH) or the original KineAssist^15^. This restricts their use to training inside rehabilitation facilities.

Wearable robotic devices like exoskeletons (e.g. Ekso, ReWalk, HAL, or X1) or exosuits^16^ provide mobile support, but are designed primarily to compensate for muscle paralysis or weakness or to decrease the metabolic cost of locomotion, and are suboptimal to support balance in users with milder impairments. In fact, most available exoskeletons, with the exceptions of bulky systems like Rex or Wandercraft, do not address balance at all and still require crutches for support.

**Figure 1:**
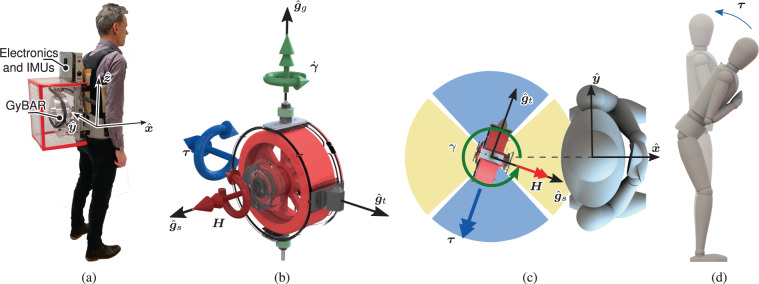
GyBAR prototype wearable gyroscopic actuator. (**a**) Model wearing the prototype GyBAR used in the experiments. (**b**) Simplified gyroscopic moment, ${\boldsymbol{\tau }}\approx -\tau {\hat{{\boldsymbol{g}}}}_{t}$, is proportional to the angular momentum, ${\boldsymbol{H}}=H{\hat{{\boldsymbol{g}}}}_{s}$, of a spinning mass and the angular rate, $\dot{{\boldsymbol{\gamma }}}=\dot{\gamma }{\hat{{\boldsymbol{g}}}}_{g}$, at which the motorized frame is gimballed (Eq. 2). (**c**) The GyBAR-fixed coordinate frame $({\hat{{\boldsymbol{g}}}}_{s},{\hat{{\boldsymbol{g}}}}_{t},{\hat{{\boldsymbol{g}}}}_{g})$ is initially aligned with the human-fixed frame $(\hat{{\boldsymbol{x}}},\hat{{\boldsymbol{y}}},\hat{{\boldsymbol{z}}})$ prior to rotation by the gimbal angle, *γ*, about the axis ${\hat{{\boldsymbol{g}}}}_{g}\Vert \hat{{\boldsymbol{z}}}$. This angle is controlled to orient ***τ*** in the direction of interest: the blue region for assistance in the sagittal plane, or the yellow region for assistance in the frontal plane. (**d**) Example use of a moment to restore upright posture in the sagittal plane.

A new type of actuation principle may enable both effective and wearable balance assistance: gyroscopic actuation. Gyroscopic actuators comprise a spinning wheel that is revolved about one or more motorized gimbal frames to produce a free moment, proportional to the rotor angular momentum and gimbal angular velocity (Fig. 1b). Earlier, we suggested that such actuators can be integrated in a backpack, providing external balance-assisting moments on a person’s body in a finely controlled way, while not even being noticeable as an aid^17^. Such a system could ultimately constitute a highly portable and wearable hands-free device that is easy to don and doff, and serve a hitherto-neglected patient group struggling with mobility due to impaired balance. Although we have recently demonstrated the efficacy of wearable gyroscopic actuators for balance perturbation^18^, only simulations^17,19–21^ and technical designs^22–24^ have been reported for the purpose of balance assistance.

We present here the first experimental investigation of gyroscopic balance assistance with human subjects, including both healthy participants and examples of potential end-users, individuals with chronic stroke. We hypothesized that moments applied to the upper body in a continuous manner would enable both groups of subjects to balance better than when unassisted. This was assessed using a prototype wearable gyroscopic actuator called *the GyBAR*^22^, which imparts gyroscopic moments on the trunk (Fig. 1a).

In a first experiment with healthy subjects (Experiment 1), we compared multiple candidate balance controllers that emulated rotational springs and dampers using only the trunk state for feedback – these gave the sensation that the body was connected in a compliant manner to a fixed frame in the sky or slowed by a viscous fluid, such as when moving through water. This virtual connection to an inertial frame, resembling overhead support systems, is only possible in this wearable system because it imparts free moments. A second, exploratory experiment (Experiment 2) gave insight into the degree to which this manner of assistance can be beneficial to a clinical population, namely individuals with chronic stroke.

**Figure 2:**
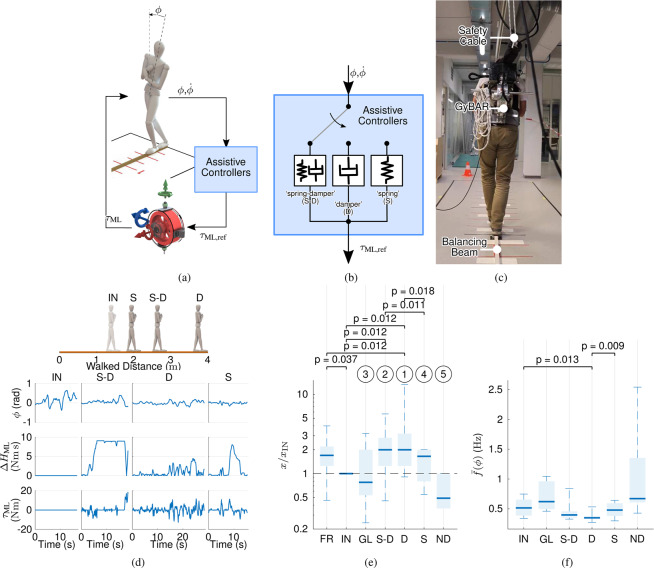
Description and main results of Experiment 1. (**a**) Schematic of balance control feedback loop. (**b**) Schematic of assistive controllers ‘spring-damper’ (S-D), ‘damper’ (D) and, ‘spring’ (S). (**c**) Subject wearing the GyBAR while traversing the beam of width 30 mm and length 4 m. (**d**) Example primary outcome measures (distance walked) and time series data for subject C13. Shown are the trunk roll angle *ϕ*, angular impulse $\Delta {H}_{ML}$, and exerted gyroscopic moment ${\tau }_{{\rm{ML}}}$ for baseline condition ‘inactive’ (IN) and assistive conditions S-D, D, and S. (**e**) Distance walked by all (*n* = 10) healthy subjects under all testing conditions, normalized to condition IN and displayed in logarithmic scale. For clarity, pairwise significance brackets (*p* < 0.05) are shown only for comparisons of controllers S-D, S, and D and conditions ‘free’ (FR, no device) and IN. Circled numbers are the perceived ‘helpfulness’ rankings, from best (1) to worst (5). (**f**) Centroidal frequency of the trunk roll angle *ϕ* for all subjects.

**Figure 3:**
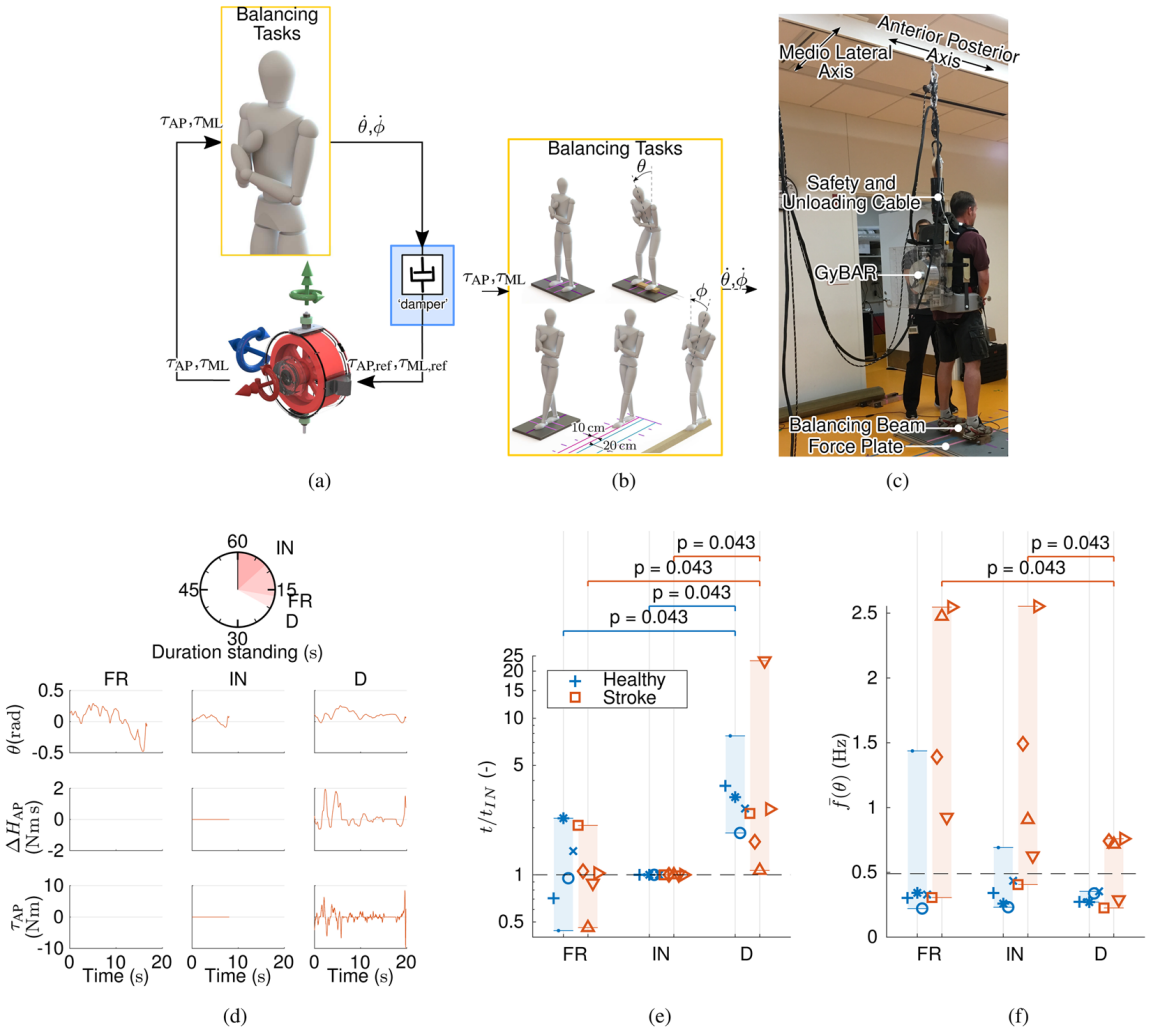
Description and main results of Experiment 2. (**a**) Illustration of ‘damper’ (D) balance controller. (**b**) Balancing tasks with reduced AP or ML bases of support. (**c**) Individual with chronic stroke wearing the GyBAR during AP balancing task over reduced BoS (100 mm). (**d**) Example primary outcomes (stance duration) and time series data for the individual with chronic stroke who exhibited the median degree of improvement with controller D, subject S1 (□). Shown are the trunk pitch angle $\theta$, angular impulse $\Delta {H}_{{\rm{AP}}}$, and exerted gyroscopic moment ${\tau }_{{\rm{AP}}}$. (**e**) Duration standing for all healthy controls (*n* = 5) and individuals with chronic stroke (*n* = 5), normalized to condition ‘inactive’ (IN) and displayed in logarithmic scale; shown are condition ‘free’ (FR) and IN and assistive controller D. Subjects H1 (+), H4 (○), and S4 (∇) all reached the maximum score in condition D. (**f**) Centroidal frequency of the trunk pitch angle $\theta$ for all subjects. The dashed line represents the median value for healthy participants in condition IN with a full BoS.

Although some definitions of balance involve continuous adaptation^25^ or robustness to perturbations^26^, we adopt here the broader functional definition as the absence of unintended contact with the ground, such as falling. Myriad measures quantifying balance have been proposed^26–28^, but their relationship with functional balance is often ambiguous or context-dependent^29–31^ and no consensus has yet been reached on a single standard. For the purposes of this study, we quantify balance using clearly interpretable functional measures derived from clinical rehabilitation, including the distance walked on a narrow beam (Experiment 1, Fig. 2c), and time stood on a narrow support surface (Experiment 2, Fig. 3c). To simplify analysis, each task challenged stability in either the frontal or sagittal plane independently. Further insights were derived from secondary measures, quantifying aspects such as the magnitude and frequency of angular motion of the trunk or the moment exerted by the actuator.

## Methods

### GyBAR as a wearable balance aid

The GyBAR is a self-contained prototype robotic balance aid that is worn on the trunk like a backpack^22^. It contains a *control moment gyroscope*, an actuator that exerts moments on the trunk by manipulating the angular momentum of an internal flywheel through rotation of a gimbal structure (Fig. 1b). This technology has been primarily developed for attitude control of spacecraft due to its ability to exert a free moment (i.e. that which does not require contact with another body or inertially-fixed frame) and potential to be lighter and more compact than comparable actuators, such as reaction wheels.

Control moment gyroscopes exert moments using the gyroscopic effect, which is induced when a spinning object changes its axis of rotation. Although variable-speed designs also exist^32^, the flywheel is typically controlled to spin at a constant angular velocity and hence have an angular momentum, ***H***, of constant magnitude, *H*. Gyroscopic moments are produced whenever a motor on the gimbal induces rotation of the flywheel with respect to the trunk (with relative angular velocity $\dot{{\boldsymbol{\gamma }}}$) or the trunk of the wearer is rotated (with angular velocity ${\boldsymbol{\omega }}={[{\omega }_{x},{\omega }_{y},{\omega }_{z}]}^{T}$, using the coordinates of Fig. 1c). A reaction moment from the gimbal motor, ${{\boldsymbol{\tau }}}_{g}$, is also exerted in the axis of the gimbal, ${\hat{{\boldsymbol{g}}}}_{g}$. The total moment applied to the trunk, 𝝉, as a function of time, *t*, is thus

1 $${\boldsymbol{\tau }}(t)=-\,\dot{{\boldsymbol{H}}}(t)=\mathop{\underbrace{-(\dot{{\boldsymbol{\gamma }}}(t)+{\boldsymbol{\omega }}(t))\times {\boldsymbol{H}}(t)}}\limits_{{\rm{g}}{\rm{y}}{\rm{r}}{\rm{o}}{\rm{s}}{\rm{c}}{\rm{o}}{\rm{p}}{\rm{i}}{\rm{c}}\,{\rm{e}}{\rm{f}}{\rm{f}}{\rm{e}}{\rm{c}}{\rm{t}}}-\mathop{\underbrace{{{\boldsymbol{\tau }}}_{g}(t)}}\limits_{{\rm{g}}{\rm{i}}{\rm{m}}{\rm{b}}{\rm{a}}{\rm{l}}\,{\rm{m}}{\rm{o}}{\rm{t}}{\rm{o}}{\rm{r}}}.$$

The moments produced by controlled motion of the gimbal $(\dot{{\boldsymbol{\gamma }}}(t)\times {\boldsymbol{H}}(t))$ are often much larger than those produced by movement of the trunk $({\boldsymbol{\omega }}(t)\times {\boldsymbol{H}}(t))$ or the gimbal motor $({{\boldsymbol{\tau }}}_{g}(t))$. Hence, ***τ*** can be approximated as

2 $${\boldsymbol{\tau }}(t)\approx -\dot{{\boldsymbol{\gamma }}}(t)\times {\boldsymbol{H}}(t)\,.$$

Expressed in the human-fixed coordinate frame $(\hat{{\boldsymbol{x}}},\hat{{\boldsymbol{y}}},\hat{{\boldsymbol{z}}})$ of Fig. 1c, this becomes

3 $${\boldsymbol{\tau }}(t)=H\dot{\gamma }(t)(\sin (\gamma (t))\hat{{\boldsymbol{x}}}-\,\cos (\gamma (t))\hat{{\boldsymbol{y}}})$$

4 $$=\,{\tau }_{ML}(t)\hat{{\boldsymbol{x}}}+{\tau }_{AP}(t)\hat{{\boldsymbol{y}}}.$$

Since generating a gyroscopic moment requires rotating the gimbal, the gimbal angle, and hence also the orientation of the gyroscopic moment, does not remain constant in the human-fixed frame. To configure the GyBAR to produce primarily frontal-plane (${\tau }_{{\rm{ML}}}$) or sagittal plane (${\tau }_{{\rm{AP}}}$) moments in this study, the gimbal angle was controlled to remain close to *π*/2 or 0, respectively, and constrained to prevent excessive off-axis moments (Fig. 1c). The gyroscopic moment was thus projected in the intended direction during nominal operation, but would cease if the maximum gimbal angle was reached. At this point, the actuator was said to be *geometrically saturated*. Further detail is provided in the supplementary information.

The GyBAR used in this study was slightly modified from that described previously^22^; it weighs 16 kg and has an estimated peak gyroscopic moment of 53 Nm. All sensors and motors interface with an on-board microcontroller (STM32-H405, Olimex, Bulgaria) and relay information via a wired RS485 connection to an off-board PC executing the high-level control loops at a fixed sampling rate of 1 kHz (implemented in Simulink Real-Time R2016b, Mathworks, USA).

### Balance-assisting controllers

Multiple candidate balance-assisting controllers were implemented, with the presumption that simple (continuous and linear) controller behaviour would be intuitive and enable users to rapidly and effectively combine the GyBAR’s support with their own balance reactions. This is inspired by evidence that humans implicitly build internal models of the world with which they interact: for example, humans are able to deduce complex quantities from ambiguous sensory input^33^ and prior experiences affect the selection of postural control strategies in response to external influences that both aid and perturb balance^34,35^. Furthermore, simple proportional or proportional-derivative feedback of kinematic states of the body have been successfully used to describe human postural control^36–39^, and might be beneficially mirrored by a biologically-inspired robotic aid.

Although relatively sophisticated estimation of the state of balance can be performed using minimal instrumentation^40^, the current study used only angular orientation and velocity of the trunk for feedback, estimated^41^ from an inertial measurement unit (IMU) located at shoulder height in the GyBAR (Fig. 1a).

Proportional, derivative, and proportional-derivative controllers were implemented, respectively equivalent to a virtual rotational spring (S, of stiffness 100 Nm/rad), damper (D, of coefficient 30 Nms/rad), and parallel spring-damper (S-D, of stiffness 100 Nm/rad and damping 30 Nms/rad) acting on the trunk. The rotational spring elements exert a moment on the trunk that guides the wearer to a nominal erect posture (calibrated at the start of each test), whereas the rotational damper element does not enforce a specific posture, but rather exerts a moment proportional to the angular velocity of the trunk, analogous to moving through a viscous medium such as water or honey. Controller gains were selected through experimentation with a healthy volunteer such that the gains were maximal yet did not lead to geometric saturation of the actuator during balance activities representative of the experimental protocol. These parameters remained fixed throughout both experiments and were not adapted per subject or task.

### Experimental protocol

Two experiments involving a different set of balancing tasks were performed: Experiment 1 compared different candidate balance-assisting controllers with healthy subjects in a walking task, and Experiment 2 explored both walking and standing balance amongst individuals with chronic stroke.

With a single gyroscopic actuator, as in the GyBAR, it is possible to assist balance in only a single axis at a time. Consequently, the protocol consisted of tasks intended to challenge sagittal and frontal balancing independently during walking or standing. These tasks induced postural instability by reducing the size of the support surface, a strategy used in a number of validated clinical tests and studies. For standing balance, this is done in the Romberg test^42^, Berg Balance Scale (BBS)^43^, Clinical Test of Sensory Interaction and Balance (CTSIB)^44^, and multiple biomechanical studies^34,45^, while walking tests with a restricted base of support (BoS) include components of the Functional Gait Assessment (FGA)^46^, Bruininks-Oseretsky Test (BOT)^47,48^, and studies involving beam-walking for balance assessment^49–51^. An advantage of this design is that it naturally suggests an unambiguous and quantifiable functional outcome measure: the distance travelled or duration remained within the allowable support surface. However this design also unintentionally constrains the efficacy or availability of primary balancing strategies, such as stepping responses or ankle moments, and therefore emphasizes other, secondary strategies, such as those involving motion of the trunk or upper limbs.

In Experiment 1, healthy subjects (*n* = 10) were asked to walk, with their feet in tandem (longitudinally aligned and with heel touching toes) and arms crossed, over a 30 mm-wide by 4 m-long wooden beam (Fig. 2) both with and without the GyBAR. Their performance was quantified as the distance between the starting location and the last point of contact of the heel with the beam. In addition to the 3 balancing-assisting controllers (S, D, S-D), 4 non-assistive conditions were tested: a deliberately perturbing negative damper (ND, −30 Nms/rad), a ‘gimbal locked’ condition in which the gimbal was fixed in place and only gyroscopic moments induced through motion of the trunk were applied (GL), an ‘inactive’ condition in which the GyBAR was worn but powered off (IN), and ‘free’ condition without the GyBAR worn (FR). All powered conditions (S, D, S-D, ND, GL) were performed 3 times each in a pre-randomized order, while the unpowered conditions (IN, FR) were performed 3 times each at both the beginning and end of the experiment (total 6 times each) to assess temporal effects such as fatigue or learning.

Experiment 2 was performed subsequently, comprising only conditions D, IN, and FR in block-random order, but involving both healthy (*n* = 5) and chronic stroke (*n* = 5) subjects. An elastic overhead suspension system (stiffness 460 N/m) was used to unload the GyBAR to an apparent weight of 7.5 kg, a load considered acceptable by clinical partners and the target mass of a subsequent GyBAR prototype. Subjects performed 3 sequences of tests separately challenging balance in either the frontal or sagittal plane during standing or walking: (i) walking on narrow beams (length 2.5 m and widths of 200 mm, 100 mm, and 80 mm), (ii) standing with feet in tandem (heel-toe) on the ground to challenge mediolateral (ML) stability, and (iii) standing with feet shoulder-width apart on wooden blocks with a small width (100 mm, 60 mm, and 40 mm) in the anteroposterior (AP) direction (Fig. 3). The walking task was scored as in Experiment 1, while both standing tasks were scored as the time until the participant moved their feet from the starting configuration, to a maximum of 120 s. Due to the disparity of balance function both between and within cohorts, each sequence was repeated multiple times with an escalating level of difficulty, wherein the width of the support surface was decreased or participants were asked to close their eyes (standing tasks only). For each participant, the easiest level in which the score ceiling (walked the entire length of the beam or remained in a stable stance for 120 s) was not reached in any of the 3 conditions was selected for analysis; the conditions are therefore compared at each subject’s respective difficulty level.

All participants wore their own athletic or low-heeled footwear. Further details of both protocols are provided in the supplementary information online.

### Subjects

For Experiment 1, 10 healthy subjects (3 female, 7 male), aged 26 to 60 years (mean age 35), were recruited with approval from the Human Research Ethics Committee of the Delft University of Technology (Study 236, August 2017). For Experiment 2, 5 individuals with chronic stroke (3 female, 2 male), aged 35 to 62 years (mean age 52) and 5 healthy controls (1 female, 4 male), aged 26 to 32 years (mean age 29), were recruited with approval from the Institutional Review Board Office of Northwestern University (Study STU00205256, October 2017). Both experiments were conducted in accordance with the recommendations of the respective review boards. All subjects were volunteers and gave their written informed consent prior to participation and for use of their data. Additional permission was received from 19 of the 20 participants to record video.

### Data acquisition

Travelled distance (walking tasks in Experiments 1 and 2) and standing duration (ML and AP standing tasks in Experiment 2) were recorded manually using floor markings and digital stopwatches. Absolute trunk angle and angular velocity were recorded at 1 kHz in Simulink from IMUs (MPU-6000 and MPU-9250, InvenSense Inc., San Jose, USA) contained within the GyBAR and located at shoulder height (Fig. 1a). In Experiment 1, the perceived ‘helpfulness’ of the random controller was indicated verbally as either ‘better’ or ‘worse’ (binary) than the preceding controller. In Experiment 2, the centre of pressure (CoP) of ground reaction forces was recorded at 1 during the standing tasks using a single force plate (Sensory Kinetics Standard, Engineering Acoustics Inc., Casselberry, USA). Video (when permitted) was used for qualitative analysis of the limb movements.

### Outcome measures

Task performance in each experimental condition was represented as the median distance (walking tasks) or duration (standing tasks) of 3 repetitions. To account for variability across subjects, these values were normalized per subject by dividing by the score at baseline condition IN, in which the GyBAR was worn but the controller turned off. This relative task performance was used as the primary outcome for statistical analysis.

Secondary outcome measures facilitated interpretation of the main findings. These included statistics computed from time series data such as root mean square (RMS), peak value, and centroidal frequency of the trunk pitch (*θ*) and roll (*ϕ*) angles measured with respect to an inertially-fixed vertical axis, trunk angular velocity ($\dot{\theta }$, $\dot{\phi }$), and centre of pressure (CoP) range and variability (standing tasks only). The relative perceived ‘helpfulness’ of each controller in Experiment 1 was converted to an absolute ranking using the Analytic Hierarchy Process^52,53^. GyBAR performance was quantified as the RMS and peak applied moment (*τ*) and angular impulse (Δ*H*, the time integral of *τ*) imparted on the wearer. Angular impulse is related to the gimbal angular displacement (Supplementary Section S1), so geometric saturation was represented as a maximum impulse Δ*H*_max_. These measures were supplemented by qualitative analysis of video footage of the experiments. Whole-body kinematics were not quantified in the present study.

### Statistical analysis

A non-parametric statistical test was selected due to the low sample size in both experiments and non-normality of the data, assessed with the signed rank Shapiro-Wilk test. A non-parametric Friedman matched samples test was performed to detect the presence of an overall effect, followed by a two-tailed Wilcoxon signed rank post-hoc test to check for pairwise significance between tested conditions, with a critical value of *α* = 0.05.

Corrections for multiple comparisons were not performed given that (i) the exploratory nature of the study prioritizes the reduction of Type II error^54–56^, (ii) the feasibility of the GyBAR for balance augmentation is restricted to the analysis of a single primary outcome measure in each experiment^56,57^, and (iii) the analysis of statistical results from additional (secondary) outcome measures were reported and interpreted only to formulate new hypotheses for further exploration^56–60^.

## Results

### Experiment 1: Effect of multiple assistive and perturbative controllers on beam walking

#### Primary outcome measures

All three assistive controllers (S-D, D, and S) increased the median distance healthy participants (*n* = 10) could walk along a 30 mm-wide (4 m-long) beam by factors of 2.0, 2.0, and 1.6, respectively, with respect to the baseline condition ‘inactive’ (Fig. 2e); however, of these, only S-D (*p* = 0.012) and D (*p* = 0.012) were statistically significant (*p* < 0.05). Despite the detriment of bearing the 16 kg mass of the prototype GyBAR (evidenced by the significant difference, *p* = 0.037, between FR and IN), controller D also showed significant improvement against even FR (*p* = 0.012).

Perturbations from self-induced gyroscopic moments during condition GL did not alter performance with respect to IN (*p* = 0.674), but intentional error augmentation with controller ND did visibly hinder balance and significantly decrease the distance subjects walked (*p* = 0.017).

No significant temporal effect (learning or fatigue) was found between the scores at the beginning and end of the experiment in conditions FR and IN (*p* = 0.123). No clear correlation was found between task performance and subject-specific parameters, such as body mass, body moment of inertia, or initial task performance.

#### Secondary outcome measures

Walking speed did not significantly change across the conditions (*p* = 0.798), and had a median value across all conditions of 0.089 m/s. Absolute trunk roll angle ${\phi }_{{\rm{RMS}}}$ and angular velocity ${\dot{\phi }}_{{\rm{RMS}}}$ were not significantly affected by any of the assistive controllers. However, a significant decrease in trunk angle centroidal frequency $\bar{f}(\phi )$ (Fig. 2f) was found for controller D ($p=0.013$).

Controller D was perceived as most helpful, followed by S-D (see ranking in Fig. 2e). Notably, controller S was perceived as worse than condition GL, although this was contradicted by superior task performance results.

The GyBAR moments $\tau$ were found to be well below the designed maximum and similar for all controllers, but the angular impulse $\varDelta H$ was considerably less conservative (Supplementary Figs. S6b,c). At least 60% of subjects encountered geometric saturation while using posture-dependent controllers S and S-D, of whom more than 70% immediately terminated the task as the assistive moment was interrupted; such saturations were observed to often follow low-frequent postural drift (e.g. prolonged leaning of the trunk), which resulted in sustained moments and a mostly monotonic exchange of angular momentum until $\Delta {H}_{{\rm{\max }}}$ was reached (Supplementary Fig. S2b). In contrast, controller D is unaffected by postural orientation: only one subject encountered geometric saturation with controller D, and continued the task without direct failure.

#### Qualitative observations

During walking and tandem stance with an unconstrained BoS, lateral balance is controlled primarily by lateral foot placement^61,62^ and moments generated by ankle eversion/inversion^62,63^, respectively. For the beam-walking task, it was visually observed that (by design) these strategies were insufficient to maintain postural stability and hence were supplemented by the greater use of a secondary strategy: dynamic manipulation of the trunk by the hip abductors/adductors to exert horizontal shear forces at the foot^34,61,64^, part of the ‘counter-rotation’ strategy in the terminology of Hof^65^. In conditions IN and ND, in which no support and perturbations were applied, respectively, this secondary trunk counter-rotation strategy was prominently visible. During baseline conditions FR and IN, 8 out of 10 participants also actively abducted/adducted their swing leg to augment balance in an apparent extension of this strategy^66^. Some subjects additionally rotated their trunk in the sagittal and longitudinal planes in complex and seemingly arbitrary patterns, which we do not interpret as useful. All such secondary strategies appeared to diminish considerably while using the assistive controllers S, D, and S-D.

### Experiment 2: Effect of damper controller in a standing balance task for both healthy and chronic stroke subjects

#### Task selection

The results of the walking and standing tasks with a reduced mediolateral (ML) BoS are not analyzed here due to (i) the prevalence of ceiling effects amongst both cohorts due to insufficient postural challenge (minimum BoS width of 80 mm) and (ii) reliance on lateral forces imparted by the overhead suspension system. This data can be found in the supplementary information online. The standing task with a reduced anteroposterior (AP) BoS, in contrast, had a low incidence of ceiling effects and the suspension system was oriented in such a way that the supporting forces were substantially decreased. These results are discussed below.

#### Primary outcome measures

Assistive controller D significantly ($p=0.043$) increased the duration that both healthy individuals (*n* = 5) and individuals with chronic stroke (*n* = 5) could remain standing on a reduced AP BoS by median factors of 3.1 and 2.5 with respect to IN (Fig. 3e).

In condition D, one individual with chronic stroke (S4 ) and two healthy subjects (H1, H4) were able to complete (i.e. achieve score ceiling) the same task that they had failed in conditions FR and IN. Those who still could not, improved their primary outcome by a median factor of 3.1 (healthy) and 2.0 (stroke). One individual with chronic stroke (S4) exhibited a substantially greater degree of improvement (23.3) than any other subject. In general, no participant exhibited a decrease in performance when the controller was turned on, although one individual subject (S3) was found to exhibit only marginal improvement (1.1); this subject already had the best within-group performance at baseline.

All subjects performed better in condition D than in IN regardless of the testing order. However, the magnitude of performance increase did appear influenced by the order in which the conditions were performed (Supplementary Table SVII), likely due to the use of block-randomization to reduce testing time. Healthy subjects typically had greater improvement with D when it was performed last (median factor of 2.25 when D first versus 3.70 when D last), while individuals with chronic stroke exhibited the opposite trend (factor of 2.63 when D first versus 1.35 when D last), suggesting that these groups may have been susceptible to slight learning and fatigue effects, respectively.

#### Secondary outcome measures

No significant differences were found between FR, IN, and D in the CoP, trunk pitch angle ($\theta$), or trunk pitch angular velocity ($\dot{\theta }$). Nevertheless, the combination of small changes in both motion range and velocity resulted in a statistically significant decrease of the centroidal frequency of both CoP ($p=0.043$) and trunk pitch angle $\theta$ ($p=0.043$) amongst the individuals with chronic stroke when controller D was active (Fig. 3f), similar to findings in Experiment 1 for frontal-plane balancing. This decrease coincided with an increase in task performance and brought the stroke group closer to the expected frequency of a healthy person in full-BoS shoulder-width stance (dashed line in Fig. 3f).

Despite the different axis of instability and user groups than in Experiment 1, the GyBAR retained similar performance characteristics. The peak and RMS gyroscopic moments $\tau$ were not substantially different between members of the healthy and chronic stroke groups, and were again considerably lower than the designed maximum (Supplementary Fig. S8c). Three subjects (H2 , H3 , and S5 ) encountered the angular impulse limit $\Delta {H}_{{\rm{\max }}}$ 4 times collectively, of which 3 immediately preceded task failure. Despite this loss of assistance, these subjects were among those who exhibited the greatest improvement in task performance when the controller was active (Fig. 3e). The actuator capabilities were sufficient for the remaining 7 subjects.

#### Qualitative observations

Sagittal balance during normal standing is maintained primarily by moments exerted by the ankle plantarflexors/dorsiflexors^67^, but a secondary dynamic hip flexion/extension strategy similar in function to that described for lateral balancing is known to occur in the case that ankle moments are insufficient or their efficacy is inhibited by a small or soft support surface^34,67^. With a reduced AP BoS, a mixture of primary and secondary balancing strategies was visually observed in both subject groups in this experiment. During condition IN, the healthy subjects exhibited persistent and high-frequent ankle plantarflexion/dorsiflexion and varying degrees of secondary hip motion; most performed the task with little or no motion of the upper body and with only minor motion of the knee joints. When controller D was active, little change in overall balancing strategy was observed amongst this group, but the frequency, and in some cases also the amplitude, of joint motions appeared to decrease.

In comparison, during all conditions, the individuals with chronic stroke exhibited clear asymmetry in the joint motions of the lower extremities (activity was visible almost exclusively on the non-paretic side) and a compensatory shifting of activity upwards, resulting in an increase of secondary hip flexion/extension and arm motion; in addition, these secondary strategies appeared to be generally more exaggerated, less coordinated with other body segments, and less consistent within and between subjects than in the healthy group. When the controller (D) was activated, a general reduction of the frequency of all joint motions was visible amongst the individuals with stroke, with compensatory secondary activity of the knees and upper extremities most noticeably reduced. Concurrently, the balance corrections at all sites appeared to be generally less random and more coordinated.

Of the stroke cohort, S3 () and S4 () had the smallest and largest task improvements in condition D, respectively, and were observed to have qualitatively different movement patterns. Subject S3 exhibited particularly low-frequent postural adjustments and each test terminated when the centre of mass slowly drifted outside of the base of support. Subject S4, in contrast, exhibited excessive and high-frequent balance reactions, which reduced when the controller was active.

## Discussion

### The GyBAR improves balance with continuous moments on the upper body

We hypothesized that continuous moments applied to the upper body would improve functional balance performance in both healthy and stroke cohorts, quantified as the normalized distance walked on a narrow beam (Experiment 1) or time standing on a block with a reduced AP BoS (Experiment 2). This was found to indeed be the case. Of the three candidate assistive controllers (‘spring’, S; ‘damper’, D; ‘spring-damper’, S-D) compared in Experiment 1 (Fig. 2e), both D and S-D significantly ($p=0.012$) increased the distance healthy subjects could walk along a beam by a median factor of 2.0 with respect to baseline condition ‘inactive’ (IN), while D also yielded significant ($p=0.012$) improvement over condition ‘free’ (FR), in which subjects were unburdened by the weight of the prototype device. Controller D yielded similar significant ($p=0.043$) degrees of improvement in Experiment 2 for a standing task (Fig. 3), in which both healthy and chronic stroke cohorts increased stance duration by median factors of 3.1 and 2.5, respectively. Controller D improved the task performance of all participants in both experiments, and enabled some subjects to reach the score ceiling of tasks that they were otherwise unable to perform.

The prescribed reduced-BoS tasks challenged balance in the AP direction during standing and in the ML direction during walking. During standing, the ground projection of the CoM is regulated within the BoS primarily by ankle and weight-shifting hip strategies^62,63,67^, while balance during walking relies primarily on a synergy of the hip abductors/adductors and foot placement of the swing leg to keep the upper body stable^61,62^. However, these balance strategies change when the BoS is reduced, in which an increased use of the dynamic hip strategy compensates for the reduced efficacy of the ankle plantarflexors/dorsiflexors and invertors/evertors during standing^34,67,68^ and greater precision of the hip abductors/adductors compensates for the limited ability of foot placement to correct for lateral motion of the CoM during the single-support phase of tandem walking^62^. Similarly, individuals with chronic stroke use the dynamic hip strategy to compensate for weakness and impaired muscle control of the affected lower limb^69^.

Despite that (or perhaps because) the GyBAR does not directly influence the motion of these joints (i.e. it is uncollocated and underactuated), it successfully complements existing balancing strategies to lead to an overall functional improvement. We presume that the effectiveness of the simple controllers investigated can be attributed in part due to the use of upper body sensory feedback, which might result in moments that mimic the dynamic hip strategies normally exhibited by healthy individuals during both standing and walking tasks and thereby contributes to better regulation of the motion of the CoM. It is also possible that simple proportional-derivative feedback terms in the GyBAR controllers mirrors the structure of the neuromotor postural controller (e.g. several validated models have the same or similar structure^36–39^), thereby simplifying how, with minimal familiarization, users of the GyBAR might integrate its controller with their own postural control system to quickly and successfully exploit these external moments. Deeper investigation is necessary to assess these explanations.

### The ‘damper’ controller decreases the frequency of postural control

For controller D, the improvement in task performance was accompanied by significant decreases in the centroidal frequency of the trunk angle with respect to condition IN in both experiments (Figs. 2f and 3f). Although significant differences between IN and D were not observed in the RMS or peak trunk angles or angular velocities, small increases in the former quantity and decreases in the latter explain the comparatively larger change in frequency. The controller opposed fast rotations and the user adapted by increasing their excursion amplitude, although it is unclear to what degree this response was an attempt to counter or to exploit the controller actions. Visual observations confirmed that, although the rate of postural corrections did appear to decrease, the influence of the controller did not lead to a qualitative change in utilized balancing strategy.

There are several possible contributing factors to the decrease in centroidal frequency of the trunk motions (Experiments 1 and 2) and CoP (Experiment 2) observed between conditions IN and D. Using models of the neuromuscular postural control system^36–39^, a decrease in frequency can be associated with an increase in sway amplitude and decrease in the stiffness of the postural control loop; this may be indicative of either decreased muscle activation in the lower limbs^70^ or decreased reflex gains^38^. A decrease in stiffness can occur when the level of postural threat is low and the high energetic cost of maintaining high stability margins is no longer justified^37,71^. Since it is unmistakable when the GyBAR is active (due to e.g. sounds and vibrations from the motors), it is conceivable that a psychological element could be present; for example, a decrease in anxiety due to a robotic placebo effect may reduce neuromotor stiffness^71–73^ – in fact, several of the individuals with stroke remarked that they felt an increase in confidence, although we did not quantify this, unlike others^74^. However, in our experiments the decrease in the frequency of postural corrections did not coincide with an increase in sway amplitude as in published observations^72,73^, hence we infer that the neuromotor control loop does not simply relax with controller D, but, rather, another mechanism exists whereby regulation of the motion of the CoM is improved such that less motor activity is required to maintain the same stability margins.

It is also possible that the GyBAR may improve stability through enhanced proprioceptive feedback, as similar differential effects have been observed between conditions of sensory deprivation and normal function (e.g.^75,76^) and between conditions of normal function and sensory augmentation (e.g. the ‘light touch’ effect^77^ and vibratory stimulation^78,79^); however, while this may contribute, it is unlikely to be the dominant reason for the improvement of task performance or reduction of sway frequency, as relatively large moments were exerted by the GyBAR on the wearer (approximately 10 Nm to 15 Nm peak, Supplementary Tables SIV and SVI).

We conclude, therefore, that our observations are primarily due to mechanical stabilization by the GyBAR, whereby the GyBAR decreased the burden of postural control such that less frequent adjustments were necessary to maintain balance.

### The ‘damper’ controller scales down balance activity

In both experiments, the reduction of the BoS resulted in a decreased ability to exert ankle moments and was compensated by a visibly greater use of secondary strategies, such as the dynamic hip strategy and manipulation of the swing leg (Experiment 1) or arms (stroke subjects in Experiment 2; in contrast, healthy participants were not permitted to use their arms for balance and only participants with chronic stroke were instructed to keep the arms as close to the body as possible).

Although not manifested in the recorded trunk angle or CoP magnitudes, visible qualitative differences in the magnitude and number of balancing strategies were observed in several subjects between conditions IN and D in both experiments. With controller D, most subjects appeared to reduce secondary balancing strategies, particularly usage of the hips, such that the primary stepping (Experiment 1) and ankle strategies were again dominant and, in some cases, almost entirely sufficient. Other secondary strategies were also observed to decrease with the controller on: swing leg exploitation in Experiment 1 appeared to cease completely, and arm motion of the individuals with stroke in Experiment 2 reduced in most cases. Subject S4 (), from the Experiment 2 stroke cohort, was originally characterized as using secondary strategies excessively, but exhibited a dramatic reduction of upper body motions and an apparent increase in coordination and effectiveness of the ankle strategy when controller D was active. Quantification of these observations should be a primary focus for subsequent study.

The reduction of secondary balancing strategies with controller D is presumed to signify greater postural stability^80^. It is well known that balancing strategies grow in magnitude and dimensionality with postural threat^34,67,68^, and modelling has shown an increase in the use of the hip strategy when postural robustness is critical^81^. Based on this model, it is deduced that visual observations of decreased hip activity with controller D is indicative of lower demand on the postural control system and greater emphasis instead on energy efficiency; however, metabolic rate was not measured in the current study, so this cannot be verified at present. In addition, it is presumed that smaller postural responses are either a consequence of smaller deviation of the CoM (however, the trunk deflection measurements, which are approximately proportional to the CoM location, do not support this) or, more likely, the CoM deviations remained somewhat consistent but postural control was shared with the GyBAR, which required less neural control. Evidence supporting the latter is the significant moment exerted by the GyBAR; however, explicit quantification of muscle activity should also be studied in the future.

### Subjects interact better with predictable controllers

It is presumed that controller D performed relatively well and was perceived better than controllers S and S-D due, in part, to a lower incidence of geometric saturations and therefore more continuous and predictable controller behavior. In the event of geometric saturation, the supporting moment would abruptly withdraw (Supplementary Fig. S2b) and, in some cases, re-engage equally abruptly, resulting in washout or a destabilizing perturbation of varying severity. In Experiment 1, more than 70% of instances in which such a situation occurred were, as a direct consequence, followed by loss of balance and task failure. Due to the high virtual stiffness of controllers S and S-D and low frequency content of human motions, controllers S and S-D resulted in a much higher exchange of angular momentum than controller D, and the angular momentum limits were encountered by an additional 50–60% of the subject group (*n* = 10, healthy).

Although the ranked perception of each controller (Fig. 2e) was influenced also by other (nominal) qualities of the controllers, the prevalence of geometric saturation often had negative consequences. This is perhaps why controller D was perceived best and controller S was perceived worse than even non-assistive condition ‘gimbal locked’ (GL), despite better task performance than GL; although GL was not found to improve balance, the stationary gimbal means that the dynamics vary less and are directly coupled to the motion of the user (i.e. no control delays or nonlinear scaling) and are therefore more predictable.

### Effective assistance does not require a reference posture

We expected that a controller regulating the trunk angle would perform best, motivated by the close relationship between angle and CoM position if inverted pendulum behaviour of postural control is assumed^37^. However, our results showed that both controllers designed to regulate posture, S-D and S, were outperformed by posture-independent controller D and, in the case of S, did not significantly improve task performance over baseline condition IN ($p=0.09$). Although this apparently contradicts our expectation, a caveat is that both controllers S-D and S were particularly prone to discontinuous (and therefore unpredictable) behavior due to geometric saturations; since this is greatly influenced by the selection of the controller gains, performed *a priori* and not adapted or ‘optimized’ per subject or task, we cannot disregard the efficacy of a posture-based control without further investigation. We can conclude, however, that it is at least feasible to assist balance without specifically accounting for postural orientation, as demonstrated with the presented implementation of controller D in both experiments.

Interestingly, the two robotic devices found to most similarly assist postural control via the upper body also used posture-independent controllers. For a walking task similar to Experiment 1 and with a wearable reaction wheel fixed to the trunk, Wojtara *et al*. implemented an anisotropic (and potentially discontinuous) damping controller to generate a velocity-dependent moment opposing motion of the trunk *only when moving away from upright posture*^82^; the selection of the viscous controller was justified with the anecdote that while ‘standing in shoulder-deep water, it is easy to keep balance’. In the second instance, Wu *et al*. used a cable robot attached to a hip harness where lateral forces proportional to the CoM velocity were applied to either assist or perturb subjects as they follow a straight line on a treadmill^35^; here the selection of the viscous field was made due to avoid imparting position constraints on the subject. Both cited studies also reported a significant improvement in task performance and positive perception when assistance was applied in this manner; it should be noted, however, that in the study of Wojtara *et al*. this comparison was made against a perturbation condition and not a neutral baseline. Although neither study discussed the selection of a damper-like controller in great detail, a separate study on performance enhancement in teleoperation has reported that interaction with a viscous field generates particularly rich haptic information of body dynamics, thereby augmenting perception and improving task performance^83^.

Virtual viscous fields also have practical benefits over their elastic and viscoelastic counterparts for human motion control. Controller D does not depend on posture and is thus robust to postural biases, asymmetries, and low-frequent drift or weight-shifting, as are common amongst individuals with stroke^69,84^; nevertheless, despite physiological differences between the post-stroke and healthy groups tested in Experiment 2, the degrees of improvement were not drastically different. We predict that a posture-independent controller of this form might also enable a wider range of tasks that specifically do require changes of posture (e.g., stooping to pick up a dropped object), both in clinical and real-world settings; it is thus of interest to further investigate the applicability of the GyBAR with such a controller in scenarios representative of activities of daily living.

Finally, arguments also exist for the suitability of certain types of controllers for this particular actuation concept. Due to a finite ability to exchange angular momentum (limited by geometric saturation), gyroscopic actuators are physically incapable of sustaining indefinite or prolonged support to overcome persistent postural bias^85^, but are rather better suited to providing dynamic assistance in response to rapid, transient motions, in which the angular momentum exchange is not monotonic but ideally varying over time such that the net exchange is small. Nevertheless, a posture-dependent controller could, in theory, be adapted to mitigate low-frequent biases, but the behaviour and perception of such a controller has not yet been studied.

### Effective assistance does not require large moments

The GyBAR exerted peak moments typically on the order of 10 Nm to 15 Nm during both experiments for both populations, considerably lower than the initial design target of 90 Nm^22^ and realized maximum capability of 53 Nm. Although the peak moment required for balance assistance depends greatly on the use-case (i.e., the degree of postural challenge experienced and the level of balance augmentation desired), it is interesting that the observed moments are lower than those previously estimated to be necessary, in the range of 25 Nm^23^ to 280 Nm^17^. These estimates assume that the user is completely passive and performs no stabilizing action of their own, yet this might be overly conservative for the majority of realistic applications, except particularly severe impairments for which such an aid might not suffice for more fundamental reasons. In fact, future design of robotic aids might benefit from even further involvement of the human user, such as by explicitly augmenting sensory feedback and thereby the user’s own balance function^86^. Further research might reveal that exploiting multiple support mechanisms (not just mechanical support) can improve the efficacy of a robotic aid, and perhaps lower the actuation requirements further. The lowest level of effective assistance might be determined through human-in-the-loop optimal selection of virtual actuation constraints and controller gains using an existing prototype^87^.

### Potential beneficiaries of the GyBAR

Our results suggest that, at least during standing balance when stepping strategies are limited, both healthy individuals and individuals with chronic stroke can improve functional performance when wearing a gyroscopic actuator on the trunk. Although further research is needed to generalize these encouraging findings to other contexts and populations, we hypothesize that the technology demonstrated with the GyBAR prototype can, in principle, be used to develop new therapy interventions potentially treating a wide range of disorders affecting balance.

Bipedal stance and locomotion are inherently unstable and rely on a number of systems for effective closed-loop control. Deterioration of the sensory system (e.g. visual, proprioceptive, and vestibular systems), nervous system (e.g. attention and coordination), or motor system (e.g. muscle weakness or paresis) can result in an inability to detect instability or correct for it sufficiently quickly and, consequently, greatly increase the risk of falling. Symptoms may be intermittent and depend on aspects such as cognitive demand during multi-tasking^88^, sensory re-weighting in changing environments^89^, or anxiety due to greater perceived risk of falling^90^. Groups with disorders affecting balance include the frail elderly (particularly those already with a history of falling)^90–92^ or individuals with cerebellar disorders^89,93,94^, vestibular disorders^38,71,95^, Parkinson’s disease^74,96,97^, or psychogenic disorders such as phobic postural vertigo^98^. Even with relatively mild impairments, it has also been suggested that fear of falling may result in delayed anticipatory postural adjustments during stepping, resulting in slower effective reaction times and greater risk of falling^99^, and stiffening of the lower limbs^72,90^. Compensatory stiffening can reduce the excursion of the CoM to improve robustness to unforeseen perturbations, but it has been argued that in certain cases this may even jeopardize stability by, e.g., (i) pushing the CoP to the edge of the BoS, causing loss of control authority^100^, (ii) decreasing the ability to react to larger disturbances in which sudden joint motion may be beneficial, such as in hip or stepping strategies^31,100^, and (iii) increasing susceptibility to resonant sway in response to high-frequency disturbances^100,101^.

For these groups, added damping by the GyBAR may be desirable for two reasons: (i) a general, pathology-nonspecific slowing of motion, and (ii) a pathology-specific compensation for insufficient neurological damping. For individuals who are unable to react quickly enough to instability, external damping can slow motion of the trunk, which may also slow motion of the centre of mass towards the edge of the base of support if the lower extremities remain relatively rigid. This would allow longer time for the individual to generate an appropriate corrective response and is presumed to explain the improved task performance and decreased frequency of motion observed in Experiment 2 for individuals with chronic stroke, who are known to have delayed and disrupted responses to stance perturbations^7^. In postural control, neurological damping has been interpreted as having an anticipatory or predictive function^102^ and is necessary to attenuate oscillatory sway that risks destabilization^38,101^. However, in old age, neurologically-generated damping has been reported to be insufficient to avoid excessive sway^101^, which may possibly be due to difficulty in modulating the time delays in muscle activity necessary to produce damping^38^. To improve robustness to sudden perturbation, the GyBAR may be able to compensate for such insufficiency. Recent research of the upper extremities has also suggested that interacting with a damping field leads to an adaptive shift of reliance from co-activation to reflex responses^103^; if this occurs also in the lower extremities, it may translate to an improved ability to react to large disturbances and execute stepping responses for persons who would otherwise rely excessively on co-activation to compensate for degraded postural control.

Thus far, the GyBAR has been investigated as an assistive device, and the tests performed gave little opportunity for adaptation. However, given the versatility of its actuation capabilities, it can potentially also be used as training device. A continuous viscous field, as investigated with controller D in this study, may be useful for bridging the transition from body-weight-supported treadmill gait training to hands-free overground training in, e.g., individuals recovering from partial spinal cord injury or stroke, by increasing confidence and mitigating risk. Other means of balance assistance are similarly possible, including an assist-as-needed approach in which the amount of support varies during motor learning^104^ or assistance that is applied only when critical to avoid a fall^105^. Previous studies have found that training with movement errors augmented by continuous force fields may improve the rate of motor adaptation of individuals with stroke^106^ or incomplete spinal cord injury^35^ during rehabilitation. Training programs based on discrete balance perturbations have been shown to reduce falls and improve reactive balance responses amongst individuals with stroke^107,108^, Parkinson’s disease^109^, and older adults^109^, while others have found such perturbations also to be useful for diagnostic purposes^31,110,111^. Although we have demonstrated both stabilizing and destabilizing fields with the GyBAR in the present study, and discrete perturbations in another^18^, no longitudinal study has yet been performed.

Some pathologies are not expected to benefit from the GyBAR. In particular, it is not the intention that the GyBAR and tested controllers can entirely replace the ability to select an appropriate balance recovery strategy following a perturbation. Rather than autonomously balancing a passive user, the GyBAR offers complementary control to temporarily delay a fall until the user can react appropriately. It may hence be unsuitable for persons with severe neurological impairments that excessively prolong or entirely obstruct balance reactions, or for persons with sensory deficits that prevent their ability to perceive instability. By design, the GyBAR is also unable to directly influence motion of the limbs, such as guide foot placement during a stepping response to a perturbation; this is in contrast to most conventional exoskeletons, which assist limb motion, but can inhibit balance. As a consequence, the GyBAR cannot independently augment the strength or weight-bearing capabilities of, e.g., persons with paraplegia, but perhaps could do so in combination with a lower-body exoskeleton, in which case the GyBAR could be worn to assist balance as the exoskeleton compensates for muscle weakness.

### Study limitations and future work

Although these findings are promising, further experimental validation is required. In particular, the results of Experiment 2 involving individuals with chronic stroke cannot be generalized due to the small sample size and high variability of the data. From this data, it is estimated that a minimum sample size of 12 subjects would be required to significantly detect a balance improvement of 100% (i.e. twice the stance time or walked distance) with a power of 80% and significance level of 5%. In addition, a direct comparison between subject groups in Experiment 2 was inhibited due to a lack of matching demographics.

The GyBAR prototype used in this study was originally constructed to explore the principles of design and control of gyroscopic actuation, and hence its 16 kg mass was not optimized for wearability or clinical usage. Indeed, excessive loading on the trunk can increases physiological strain^112^ and is counterproductive to the goal of improving balance^113,114^. The latter is illustrated in Experiment 1, where two of the three assistive controllers were not found to be statistically better than simply removing the device (Fig. 2e). However, unloading the GyBAR to an apparent 7.5 kg in Experiment 2 appeared to eliminate this effect. We are currently developing a significantly lighter second GyBAR targeted towards clinical use. This will retain similar performance characteristics but have a more ergonomic attachment to the body (Supplementary Fig. S4) and a lower mass (9 kg), achieved by using a lighter rotor but increasing its rate of rotation (Supplementary Section S3.2). Reducing the actuation requirements can further reduce the mass and size of future prototypes, but the lowest level of effective assistance must first be determined.

Greater understanding of any underlying neurological adaptations during use of the GyBAR requires additional outcome measures to be recorded. Optical motion capture can give insight into the limb and centre of mass kinematics and enable the computation of more sensitive measures of stability, e.g. involving the extrapolated centre of mass^115^, or elaborate on our observations of decreasing movement frequency of the trunk and limbs. Electromyography of the lower extremities may also reveal whether subjects change their movement strategies when using the GyBAR, and the degree to which performance changes are due to mechanical support, psychological factors (e.g., increased confidence or elevated alertness), or sensory augmentation. Quantifying either perceived^116^ or metabolic effort during prolonged usage may be beneficial for refining the design requirements and use-case of more mature prototypes.

For ease of interpretation, our study evaluated a limited set of balancing tasks with simple outcome metrics based on validated clinical balance characterization tests. However, these somewhat artificial activities do not fully encompass the envisioned potential of the GyBAR concept for overground support in multiple environments, including the home and outdoors, or for a spectrum of activities of daily living. It is therefore of great interest to investigate additional, more realistic and complex examples of activities in which other balance recovery strategies might be employed, such as stepping to increase the base of support, or in which stability is perturbed through other means, such as slipping, tripping, or pushing.

## Conclusion

It was found that gyroscopic moments applied to the upper body can significantly improve functional balance during both standing and walking amongst healthy subjects. Notably, the most successful and best-perceived candidate assistive controller did not require a reference posture and was able to reduce the frequency of motion of the trunk. An exploratory investigation found similar promising results amongst individuals with chronic stroke, suggesting potential utility for this technology in rehabilitation. Further research is recommended in order to generalize these findings and increase the scope to other target groups and use-cases. Subsequent study should consider greater analysis of the underlying neurological processes to further explain the reasons for these balance improvements.

## Supplementary information

Supplementary Information.
