# Supplementary material for: Controller synthesis and clinical exploration of wearable gyroscopic actuators to support human balance
Source: Sci Rep. 2020 Jun 26;10:10412. doi: 10.1038/s41598-020-66760-w (PMC7320159; doi:10.1038/s41598-020-66760-w)
Supplement: Supplementary file 1 — Supplementary Information. [file 41598_2020_66760_MOESM1_ESM.pdf]

Controller synthesis and clinical exploration of wearable gyroscopic actuators to support human balance

Daniel Lemus[1,†], **Andrew Berry**[1,†], **Saher Jabeen**[1], **Chandrasekaran Jayaraman**[2], **Kristen Hohl**[2], **Frans C. T. van der Helm**[1], **Arun Jayaraman**[2], **and Heike Vallery**[1,*]

[1]Department of Biomechanical Engineering, Delft University of Technology, Delft, 2628 CD, The Netherlands
[2]Max Näder Center for Rehabilitation Technologies & Outcomes Research, Shirley Ryan AbilityLab, Chicago, IL 60611, U.S.A.
[†]A.B. and D.L. have contributed equally.
[*]Corresponding author: h.vallery@tudelft.nl.

ABSTRACT

This supplementary document contains an explanation of the GyBAR principles of operation, greater detail of the experimental protocol, additional results, and technical discussion points not included in the main article.

Supplementary Material

S1 Gyroscopic actuation

S1.1 Gyroscopic moment

The GyBAR contains a control moment gyroscope, which consists of a spinning rotor fixed to a motorized gimbal frame (Supplementary Fig. S1a). The gyroscopic moment applied to the person has magnitude proportional to the gimbal angular velocity $\dot{\boldsymbol{\gamma}}(t) \in \mathbb{R}^3$, the angular velocity of the person $\boldsymbol{\omega}(t) \in \mathbb{R}^3$, and the angular momentum of the rotor $\boldsymbol{H}(t) = \boldsymbol{H}(\boldsymbol{\gamma}(t)) \in \mathbb{R}^3 \mid H = \|\boldsymbol{H}(t)\| = J\Omega$, where J and Ω are the respective spin-axis moment of inertia and angular velocity of the rotor (assumed constant). The total GyBAR moment $\boldsymbol{\tau}(t) \in \mathbb{R}^3$ also includes a reaction moment from the gimbal motor, $\boldsymbol{\tau}_g(t) \in \mathbb{R}^3$. This moment can be decomposed into two components, one of which results from controlled motion of the gimbal and that the other is uncontrolled and induced by motion of the person:

$$\boldsymbol{\tau}(t) = -\dot{\boldsymbol{H}}(t) \tag{S1}$$

$$= -\underbrace{(\dot{\boldsymbol{\gamma}}(t) + \boldsymbol{\omega}(t)) \times \boldsymbol{H}(t)}_{\text{gyroscopic effect}} - \underbrace{\boldsymbol{\tau}_g(t)}_{\text{gimbal motor}} \tag{S2}$$

$$= -\underbrace{\left(\dot{\boldsymbol{\gamma}}(t) \times \boldsymbol{H}(t) + \boldsymbol{\tau}_g(t)\right)}_{\text{controlled}} - \underbrace{\boldsymbol{\omega}(t) \times \boldsymbol{H}(t)}_{\text{uncontrolled}} . \tag{S3}$$

When $\boldsymbol{\tau}_g$ and $\boldsymbol{\omega}$ are considered small in comparison to $\dot{\boldsymbol{\gamma}}$ and are neglected, $\boldsymbol{\tau}$ is oriented opposing the gimbal-fixed $\hat{\boldsymbol{g}}_t$ axis shown in Supplementary Fig. S1a. In Experiment 1, the effects of the uncontrolled term were isolated with the condition 'gimbal locked' (GL, Supplementary Table SII), which was found to not significantly impact beam-walking performance compared to the condition 'inactive' (IN), in which $\|\boldsymbol{H}\|$ is zero; see Supplementary Fig. S5.

The gimbal-fixed coordinates map to a human-fixed coordinate system via rotations by the gimbal angle γ about the $\hat{\boldsymbol{g}}_g$ axis. For a human-fixed frame $(\hat{\boldsymbol{x}}, \hat{\boldsymbol{y}}, \hat{\boldsymbol{z}})$ oriented as *(anterior,left,up)*, and with a vertical gimbal axis (as in this study), the gyroscopic moment spans the axial plane:

$$\boldsymbol{\tau}(t) \approx -\dot{\boldsymbol{\gamma}}(t) \times \boldsymbol{H}(t) \tag{S4}$$

$$= J\Omega\dot{\gamma}(t)\left(\sin\left(\gamma(t)\right)\hat{\boldsymbol{x}} - \cos\left(\gamma(t)\right)\hat{\boldsymbol{y}}\right) \tag{S5}$$

$$= \tau_{\mathrm{ML}}(\gamma(t), \dot{\gamma}(t))\,\hat{\boldsymbol{x}} + \tau_{\mathrm{AP}}(\gamma(t), \dot{\gamma}(t))\,\hat{\boldsymbol{y}} \tag{S6}$$

where non-bold symbols are scalar quantities with signs as defined in Supplementary Fig. S1a.

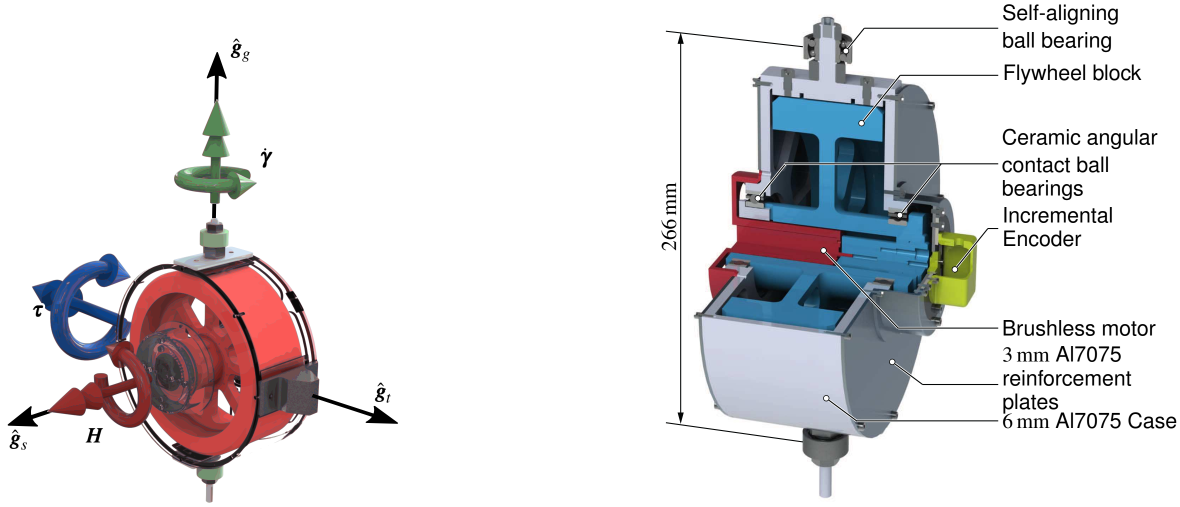

(a) **(b)**

Figure S1. GyBAR schematics. (a) Dynamics of the GyBAR showing the orientation of the angular momentum vector ($\boldsymbol{H}$), gimbal rotation ($\dot{\boldsymbol{\gamma}}$), and output gyroscopic moment ($\boldsymbol{\tau}$, shown for the case that the trunk angular velocity, $\boldsymbol{\omega}$, is small in comparison to $\dot{\boldsymbol{\gamma}}$). (b) Section view of the prototype GyBAR actuator and its components (gimbal motor not shown). Taken with permission from[1].

S1.2 Angular momentum exchange and geometric saturation

Gyroscopic actuators cannot indefinitely exert a moment (or moment component) in a fixed direction. Since generating a gyroscopic moment requires rotating the gimbal (with angular rate $\dot{\boldsymbol{\gamma}}$), the projection of $\boldsymbol{\tau}$ onto the human frame (which depends on γ) will vary with time. For a reference moment fixed statically in the human frame, the gimbal will thus ultimately rotate to a configuration in which either mechanical limitations prohibit it from moving further (as in this study) or $\boldsymbol{\tau}$ becomes orthogonal to the reference; both instances are referred to here as *geometric saturation*, while the latter is referred to more specifically as a *geometric singularity*.

It is possible to express the proximity to geometric saturation using the angular momentum state vector as a function of the gimbal angle, $\boldsymbol{H}(\gamma(t))$. This has the advantages that (i) actuator performance can be expressed in terms of an important design variable, the rotor angular momentum magnitude H, and (ii) a relationship to the generated moment can be found by integrating Supplementary Eq. S1. For any time t, the angular impulse between the rotor and person, $\Delta\boldsymbol{H}(t)$, can be expressed as:

$$\Delta\boldsymbol{H}(t) = \boldsymbol{H}(t) - \boldsymbol{H}(0) = \int_0^t \dot{\boldsymbol{H}}(T)\mathrm{d}T = -\int_0^t \boldsymbol{\tau}(T)\mathrm{d}T \tag{S7}$$

The angular momentum state $\boldsymbol{H}(t) = \boldsymbol{H}(\gamma(t))$ can change only through rotation of the gimbal and hence prescribes an arc of radius H (or a circle, if γ is unconstrained). As a result, the exchanged momentum $\Delta\boldsymbol{H}(t)$ is bounded to a maximum of $2H$, although this can be smaller depending on the initial state $\boldsymbol{H}(0)$ in relation to the direction of $\boldsymbol{\tau}$ and the presence of further limitations on γ. Equivalently, this acts as a constraint on the time integral of $\boldsymbol{\tau}$. It is therefore pertinent to measure the trajectory of $\Delta\boldsymbol{H}(t)$ to determine how close the actuator comes to geometric saturation and quantify overall performance.

As in Supplementary Eq. S6, $\Delta\boldsymbol{H}$ can be decomposed into human-fixed coordinates:

$$\Delta\boldsymbol{H}(t) = \underbrace{H\left(\cos\gamma(t) - \cos\gamma(0)\right)}_{\Delta H_{\mathrm{ML}}}\hat{\boldsymbol{x}} + \underbrace{H\left(\sin\gamma(t) - \sin\gamma(0)\right)}_{\Delta H_{\mathrm{AP}}}\hat{\boldsymbol{y}} \tag{S8}$$

where γ is defined such that $\boldsymbol{H}||\hat{\boldsymbol{x}}$ when $\gamma = 0$. For compactness of notation, the term ΔH is used to mean either one of these scalar components, depending on the context. Supplementary Fig. S2 illustrates the phenomenon and consequences of geometric saturation in the context of human balance control; the angular momentum exchange reaches a maximum when the gimbal reaches its mechanical end-stop, causing the gyroscopic moment to cease.

Given the importance of the angular momentum of the rotor H for both the magnitude and duration of a gyroscopic moment (Supplementary Eq. S3 and S7, respectively), it is a fundamental design parameter for gyroscopic actuators. For a fixed $H = J\Omega$, Supplementary Fig. S3 shows the tradeoff between mass and size (both relating to rotor moment of inertia J) and rotor speed Ω during GyBAR design optimization.

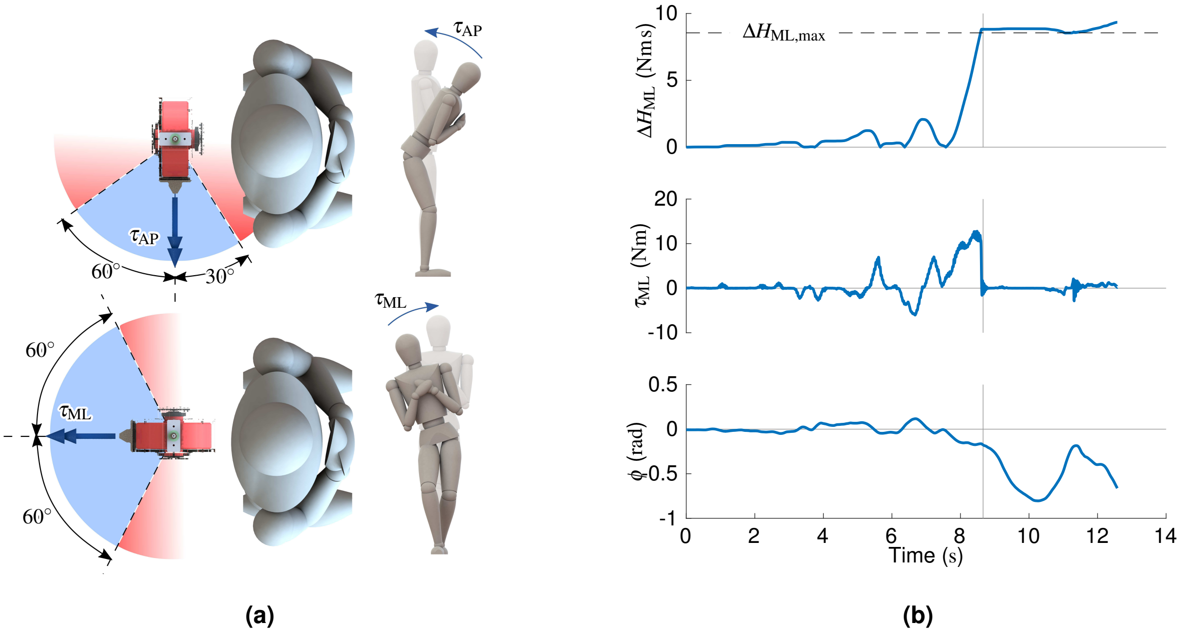

Figure S2. Geometric saturations. (a) Overhead views showing the ranges of gimbal operation (in blue) for AP (top) and ML (bottom) moment (τ) generation. When the gimbal rotates such that τ approaches the boundary, the gimbal is arrested and moment generation ceases, referred to here as *geometric saturation* of the gyroscopic actuator. (b) Example timeseries data of subject C14 of Experiment 1, showing the occurrence of geometric saturation (vertical line) with the 'spring' (S) controller. As the subject leans (ϕ), the controller produces an opposing moment (τ); the gimbal reaches its limit when the time integral of the moment (the exchanged angular momentum in this direction, ΔH_{ML}) reaches a maximum ($\Delta H_{ML,max}$), at which point τ ceases.

S1.3 The GyBAR

The GyBAR contains a single control moment gyroscope (CMG), which shown in Supplementary Fig. S1b and has been described in detail previously[1].

In most applications, arrangements of multiple CMGs are used to control both the magnitude and direction of the output moment simultaneously and, if a redundant number is available, also provide options for avoiding singularities[2]. However, the first GyBAR prototype consists of only a single CMG and the direction of the gyroscopic moment is not independently manipulable. In the present study, the gyroscopic moment was projected in approximately the intended direction during nominal operation by restricting the gimbal angle. Due to this constraint, in addition to further gimbal restrictions to avoid cable entanglement, the estimated maximum moment was reduced to ± 43 Nm for balancing in the frontal plane (Experiment 1) and -32 Nm to 43 Nm in sagittal plane (Experiment 2).

The GyBAR was modified for this study from the version described previously[1]. For the present study, the GyBAR gimbal motor and gearbox were replaced to reduce weight; this change reduced the mass by almost 1 kg, at the cost of a reduction of estimated peak gyroscopic moment from 70 Nm to 53 Nm.

S2 Experiment protocol

S2.1 Experiment 1

Ten healthy adults (3 female, 7 male) of various age (mean 34.7 years, standard deviation 11.6 years) and without known balance impairment were recruited for Experiment 1 (Supplementary Table SI). Subjects were asked to walk, with feet in tandem (i.e. feet aligned laterally and with the heel of the anterior foot touching the toes of the posterior foot during the double-support phase of the gait cycle) and with their arms crossed, over a 3 cm wide by 4 m long wooden beam, both with and without wearing the GyBAR. Such beam-walking tests have previously been explored for characterizing the balance performance of amputees[3].

During each test, an overhead rail provided emergency fall prevention by means of slack ropes fixed to both a safety harness worn by the participant and the GyBAR itself; care was taken to ensure that neither rope was under tension at any time, such that they could not be exploited for mechanical stabilization.

Seven different conditions were tested, as listed in Supplementary Table SII. Due to the time needed to don/doff the device and accelerate/decelerate the rotor to/from its nominal speed, the conditions were block-randomized for efficiency. Conditions 'free' (FR) and 'inactive' (IN) both preceded and succeeded a block-randomized set of the conditions in which the GyBAR was both worn and active ('gimbal locked', GL; 'spring', S; 'damper', D; 'spring-damper', S-D; and 'negative damper', ND; referred to here as 'device active' conditions). The experiment was thus performed as follows, with three repetitions per condition:

1. Baseline condition 'free' (FR): x3 repetitions in succession.
2. Baseline condition 'inactive' (IN): x3 repetitions in succession.
3. Randomized 'device active' conditions (GL, S, D, S-D, ND): x3 repetitions each in fully random order.
4. Baseline condition 'inactive' (IN): x3 repetitions in succession.
5. Baseline condition 'free' (FR): x3 repetitions in succession.

The task was terminated when the subject either (i) reached the end of the beam, (ii) contacted the ground beneath the beam, (iii) violated the 'tandem stepping' constraint, (iv) violated the 'crossed arms' constraint, or (v) relied on either of the safety ropes for assistance; in the rare event of (iii-v), the subject was asked to repeat the condition. The primary outcome measure was the distance walked along the beam until task termination, measured as the last point of contact of the heel on the beam. In addition, task duration (recorded from the moment of first foot placement on the beam) was recorded manually, and trunk angle and angular velocity were measured using inertial measurement units (MPU-9250, InvenSense Inc., San Jose, CA, USA, at 1000 Hz) embedded in the GyBAR ('inactive' and 'device active' conditions only) and a nonlinear state estimator[4]. During the randomized 'device active' conditions, subjects were also asked after each task whether they felt the helpfulness of the controller was either 'better' or 'worse' than that in the previous task; only binary responses were accepted. The data was condensed by comparing only the results of the trial resulting in the median distance walked of the three repetitions per condition and per subject.

S2.2 Experiment 2

In Experiment 2, five stroke survivors with mild balance impairments and an equal number of healthy control subjects were recruited (Supplementary Table SI, note that no effort was made to match the ages of the groups). To participate in the study, both post-stroke (S) and healthy (H) participants had to meet the following inclusion and exclusion criteria:

Inclusion criteria

- (S,H) Must be able and willing to give written consent and comply with study procedures.
- (S,H) Must be between 18-85 years of age.
- (S,H) Must be able to fit in the device, with chest measurements in the range 98-132 cm and waist measurements in the range 80-150 cm.
- (S,H) Must be between 1.50 m and 2.0 m in height (flexible requirement).
- (S,H) Weigh between 50 kg to 100 kg (flexible requirement).
- (S,H) Must be able to tolerate upright standing position with device 7.5 kg for 30 min.
- (S,H) Must be able to ambulate 10 m without physical assistance or use of an assistive device (ankle orthoses and braces permitted).
- (S) Must be greater than 6 months post stroke.
- (S) Must be able to perform unsupported Romberg balance on firm surface with eyes open for a minimum of 30 s.
- (S) Must demonstrate balance deficit as measured on condition 2, 3, or 4 of the modified Clinical Test of Sensory Interaction on Balance (mCTSIB) in Romberg (feet together) or tandem (one foot directly in front of the other foot, heel touching toe) position of less than 30 s average.

Table SI. Participant information for both experiments.

Subject	Marker	Group[1]	Gender	Age	Mass (kg)	Height (m)	Stroke type[2]	Years since stroke	Dominance[3]	Hemiparesis	BBS[4]	FGA[5]	mCTSIB[6]
**Experiment 1**													
C05			F	26	70	1.68							
C06			M	35	91	1.82							
C07			F	26	57.5	1.66							
C08			M	60	86	1.91							
C09			M	28	82.3	1.83							
C10			M	34	73	1.79							
C12			F	51	80	1.73							
C13			M	27	70	1.83							
C14			M	30	78	1.86							
C15			M	30	95	1.94							
**Experiment 2**													
S1	□	B	M	49	79	1.83	IS	5.5	R	R	53	22	[30,30,9,5]
S2	◇	A	M	57	101	1.83	IS	1.3	R	L	53	22	[11,7,-,-]
S3	△	A	F	35	70	1.80	HS	1.1	R	R	52	23	[30,26,21,5]
S4	▽	B	F	62	64	1.63	IS	5.0	R	L	42	13	[30,3,16,2]
S5	▷	B	F	59	88	1.65	HS	9.4	R	R	48	16	[16,9,-,-]
H1	+	A	M	32	91	1.91							
H2	•	A	F	30	64	1.75							
H3	∗	A	M	27	73	1.83							
H4	○	B	M	26	73	1.83							
H5	×	B	M	29	70	1.70							

1 Group testing orders. Group A: free-inactive-damper (FR-IN-D). Group B: free-damper-inactive (FR-D-IN).
2 IS: Ischemic, HS: Hemorrhagic.
3 Side of dominance identified as the side of preference for writing.
4 Berg Balance Scale[5]: to a maximum of 56 points.
5 Functional Gait Assessment[6]: to a maximum of 30 points.
6 Modified Clinical Test of Sensory Interaction on Balance[7]: to a maximum of 30 s per item (average of three trials). Reported values are for tandem stance; values for Romberg position were 30 s for all participants for all items. Subjects S2 and S5 were unable to assume tandem stance in the last two tasks.

Table SII. Experiment 1 description of controllers and conditions.

			Balance Controller Gains[1]	
Condition	Abbrv.	Description	K_p $\mathrm{N\,m\,rad^{-1}}$	K_d $\mathrm{N\,m\,s\,rad^{-1}}$
‘free’	FR	Device is not worn. Subjects wear only a loose safety harness.	-	-
‘inactive’	IN	Device is worn but power off, acting as dead weight. Here the effect of added mass on task performance is tested, serving also as baseline for the conditions in which the device is worn.	-	-
‘gimbal locked’	GL	Device is worn with rotor spinning, but controller off. Gimbal position is kept in place to test the effect of self-induced, or *parasitic*, moments).	-	-
‘spring-damper’	S-D	Device is worn and active with an assistive controller, consisting of a continuous visco-elastic field (rotational spring and damper). Controller is intended to maintain a user-determined reference posture, while reducing sudden torso movements.	100	30
‘damper’	D	Device is worn and active with an assistive controller, consisting of a continuous viscous field (rotational damper). Controller is intended to reduce sudden torso movements without affecting subject’s posture.	0	30
‘spring’	S	Device is worn and active with an assistive controller, consisting of a continuous elastic field (rotational spring). Controller is intended to maintain a user-determined reference posture.	100	0
‘negative damper’	ND	Device is worn and active with an error-augmenting controller, consisting of a continuous additive viscous field (negative rotational damper). Controller augments sudden torso movements.	0	-30

[1] K_p: proportional gain (rotational spring stiffness). K_d: derivative gain (rotational damper viscosity).

- (S) Mini Mental Status Exam (MMSE) score > 17.
- (S) Medical clearance from physician.

Exclusion Criteria

- (S,H) Currently pregnant (self-reported).
- (S) Medical issues that impede the patient from carrying the full body weight and ambulation.
- (S) Cognitive and/or communicative disability (e.g. due to brain injury). Subjects must be able to follow directions and communicate their experiences to the researchers.
- (S) Untreated deep vein thrombosis (DVT).
- (H) Previously diagnosed balance impairment.

In addition to this, the post-stroke group underwent a baseline functional balance assessment consisting of the Berg Balance Scale (BBS)[5], Functional Gait Assessment (FGA)[6], and the modified Clinical Test of Sensory Interaction on Balance (mCTSIB)[7], all of which were performed without wearing the GyBAR; the results are presented in Supplementary Table SI.

The experimental protocol consisted of three sequences of balancing tests with increasing difficulty that challenged either mediolateral (ML) or anteroposterior (AP) balancing by constraining the size of the support surface: (i) walking heel-toe between two lines or on a beam (similar to Experiment 1), (ii) standing with feet in tandem, (iii) standing with feet shoulder-width apart on a surface with decreased AP base of support (BoS). The ML and AP directions of instability were challenged separately because the prototype GyBAR is not currently capable of influencing balance in both axes simultaneously. Nevertheless, the chosen balancing tasks do not deviate far from validated clinical tests and peer-reviewed studies: the standing tests are similar to those used in the Romberg test[8], BBS[5], and CTSIB[7], the use of a reduced BoS in AP balance is inspired by work by Horak and Nashner[9], and walking tasks with narrow bases of support have been used in the FGA[6], Bruininks-Oseretsky Test (BOT)[10,11], and in the works of Sawers *et al.*[3,12]. This protocol was performed with a subset of the conditions from Experiment 1: 'free', 'inactive', and 'damper' (Supplementary Table SII).

In the walking tasks, subjects were instructed to walk heel-toe 2.5 m between two lines set 20 cm or 10 cm, or along a foam gymnastics beam of the same length and 8 cm width; as in Experiment 1, subjects were instructed to keep their arms close to their bodies, and total distance walked before violation of the prescribed constraints was recorded as the primary outcome measure. In the standing task with a reduced ML base of support, subjects were instructed to stand with their feet again in a tandem heel-toe position, but refrain from shifting their feet for 120 s; the primary outcome measure was the test duration up to the point that (i) 120 s had elapsed, (ii) the feet moved from their initial configuration, (iii) the 'arms crossed' constraint was violated[1], or (iv) the cable holding the safety harness was taut and fully extended. In the standing task with a reduced AP base of support, the same procedure was followed, but with the feet parallel and shoulder-width apart (specifically, the medial edges of both feet were separated by 20 cm) and while standing on support surfaces of different AP dimension.

In order to account for differences in subject balance function and reduce the occurrence of ceiling effects, the challenge of each sequence was progressively increased by reducing the BoS or depriving the subject of vision; the exact construction of each sequence is described in Supplementary Table SIII. If a task could be completed (score ceiling attained) in the first attempt, the subject would progress immediately to the next task within the sequence; otherwise, they would repeat the same task three times and progress through the sequence no further than the next task. To preserve this graduated structure within each sequence and reduce the time and inconvenience of switching between active and inactive conditions, each sequence was performed uninterrupted and only the order of conditions 'inactive' and 'damper' was randomized.

Whereas all participants in Experiment 1 were healthy and capable of bearing the mass of the prototype GyBAR (16 kg), in Experiment 2 the different load-bearing capabilities of the stroke survivors were accommodated by unloading part of the added weight. In conditions 'inactive' and 'damper', a soft spring (460 N/m) connected the GyBAR to a two-axis gantry ceiling lift system (Fig. 3c in main document) and unloaded the GyBAR to an effective weight of 7.5 kg (note, however, that the inertia of the device could not be compensated for). The apparatus was arranged such that walking or anteroposterior motions occurred in the axis of the gantry system with the lowest amount of inertia; the second axis had comparably high inertia and could be considered to be approximately fixed.

As in Experiment 1, task-based performance metrics (distance walked or duration stood) were used as the primary outcome measures, and trunk angle and angular velocity were used as secondary measures. In addition, standing tests were performed on top of a single forceplate (Sensory Kinetics Standard, Engineering Acoustics, Inc., Casselberry, FL, USA, at 100 Hz). The hardest task in each sequence that was attempted in all testing conditions was selected as the basis for comparison for that subject, and, in the instance of multiple trials per condition, the trial with the median primary outcome score was selected.

[1]This constraint was only verbally enforced for the post-stroke group, who often had difficulty achieving or maintaining the specified arm configuration.

Table SIII. Experiment 2 tasks description.

Task	BoS[1]cm	Eyes	Basis for Comparison[2]
**Walking (constrained ML BoS)[3]**			
Virtual beam	20	Open	
Virtual beam	10	Open	S4
Foam beam	8	Open	S1-3, S5, H1-5
**Standing (constrained ML BoS)[4]**			
Static stance	full	Open	
Static stance	full	Closed	S1-5, H1-5
**Standing (constrained AP BoS)[5]**			
Flat surface	full	Open	
Flat surface	full	Closed	
Wooden block	10	Open	
Wooden block	10	Closed	
Wooden block	6	Open	S1-2, S4-5
Wooden block	6	Closed	S3
Wooden block	4	Open	H1, H3-5
Wooden block	4	Closed	H2

1 Base of Support size in constrained direction.
2 The test selected for comparison for each subject was the most difficult in which all three conditions were attempted. After failing to achieve the maximum score on a test, subjects were permitted to attempt only one level of difficulty higher.
3 Tandem walking over a 2.5 m beam. BoS is width of delineated virtual beam or foam gymnastics beam.
4 Tandem stance on a flat surface with no BoS restriction.
5 Feet placed hip-width apart. BoS is standing on either flat ground or a wooden block with toes and heels overhanging.

S3 Technical guidelines

S3.1 Effective controllers can be realized with simple instrumentation

Despite the simplicity of the controllers investigated, controllers D and S-D were found to be effective for augmenting balance function. All controllers used sensors located only within the GyBAR to limit inconvenience when donning and doffing the device. Implementation of controller D is particularly simple, as the output of a gyroscopic sensor can directly be used for feedback, without the computational complexity of postural state estimation via fusion of different sensor types in an inertial measurement unit.

S3.2 Peak level of assistance can be traded for mass reduction

Although the GyBAR provided sufficient assistance to improve balance, its weight is impractical for potential target groups such as recovering stroke patients or the frail elderly. The 16 kg weight of the GyBAR, corresponding to between 15-28 % of the bodyweight of the participants of both experiments (Supplementary Table SI), was found to have a significant detrimental effect on the balance of even young healthy individuals, as evidenced by the significant difference between the 'free' (FR) and 'inactive' (IN) conditions in Experiment 1 (Fig. 2e, p=0.037), and was observed to result in increased spine flexion and neck extension during standing. Physiological studies on backpack loading suggest that, in order to avoid increased exertion or perturbed posture, backpack-type loads should not exceed 20-30 % bodyweight in young healthy individuals[13, 14]; however, other factors should also be considered, such as the duration of load carriage, construction of the backpack (including distribution of the load and concentrations of pressure), and the physical condition of the wearer[13], all of which may affect how the body is strained[15]. For impaired or frail target populations, it is expected that the maximum load should be much smaller; in Experiment 2, for example, the device when unloaded to 7-13 % bodyweight appeared to be acceptable for short durations for individuals with chronic stroke, yet specific investigation of these thresholds is required.

In the design of gyroscopic actuators, a trade-off exists between performance, size, mass, and rotor speed. Both the maximum gyroscopic moment, τ, and the angular momentum exchange, ΔH, are proportional to the (constant) angular momentum magnitude of the rotor, $H{=}J\Omega$, where Ω is the rotor spin-axis angular velocity and J is the rotor moment of inertia, which is itself proportional to the mass and square of the radius of the rotor. Thus, for the same rotor angular momentum (i.e. similar performance specifications), it is possible to reduce the rotor mass by increasing its speed and/or radius. For the same $H{=}10\,\mathrm{N\,m\,s}$ and size of the GyBAR in this study, a new device of 7.5 kg mass (approximately 10 % body weight), as emulated in Experiment 2, could be realized by increasing the rotor speed from 5000 to 9000 rpm (Supplementary Fig. S3); this higher speed could be achieved by incorporating friction-reducing components such as specialized bearings and/or a low-pressure rotor chamber.

Our results suggest that only a fraction of the maximum capabilities of the GyBAR were exploited by median users: less than the 35 % of the maximum gyroscopic moment and less than 60 % of the exchangeable angular momentum. It would hence be possible to reduce the rotor angular momentum in proportion, meaning, for example, that a new device might have less mass even if the size and speed were to remain unchanged. However, our findings indicate that encountering geometric saturation (i.e. exchanging the maximum amount of angular momentum) is particularly detrimental to balance performance and should be avoided with a safety margin similar to that realized in this study. Finally, it is important to note that these performance specifications will, in general, depend on (i) the baseline balance function of an individual, (ii) the degree of postural challenge expected, and (iii) the controller gains and intended degree of balance improvement; further experimentation may thus be necessary to optimize the mass of the device for other groups or applications.

S3.3 Handling of geometric saturations will improve performance

Despite being less problematic in posture-independent controller D, further precautions should be taken to reduce the frequency or severity of perturbations resulting from geometric saturation.

Although geometric saturations are inherent limitations of gyroscopic actuators, the frequency at which saturation occurs can be reduced by (i) increasing the angular momentum of the rotor or adding additional rotors to a similar cumulative effect, (ii) modifying the controller to exert less moment (e.g. reduce the spring or damper gains) or be less sensitive to biases or low-frequent dynamics, or (iii) increasing the degree to which the available angular momentum can be exchanged. Increasing the angular momentum as in (i) presents a trade-off with increasing the mass of the device, but is feasible to the extent discussed previously. Reducing controller sensitivity to biases as in (ii) can be accomplished by selecting different feedback signals, high-pass filtering these signals, or designing a supervisor that regulates the nominal projection of the angular momentum. Finally, (iii) is typically achieved by coordinating two or more (possibly smaller) gyroscopic actuators such that the net gyroscopic moments are projected in the desired direction, either via model-based control[2, 16] or a mechanical constraint[17, 18]; the presented GyBAR prototype was further constrained by the presence of electrical cables that prevented the gimbal from rotating freely, which can be replaced in the future with slip-rings.

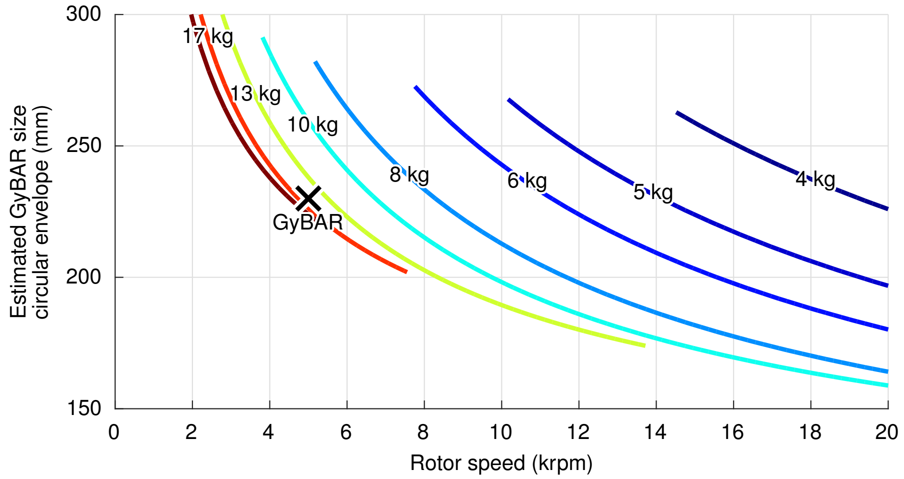

Figure S3. Approximated GyBAR size and mass (iso-lines) as a function of rotor angular velocity Ω for the performance of the current prototype ($H = 10\,\mathrm{N\,m\,s}$).

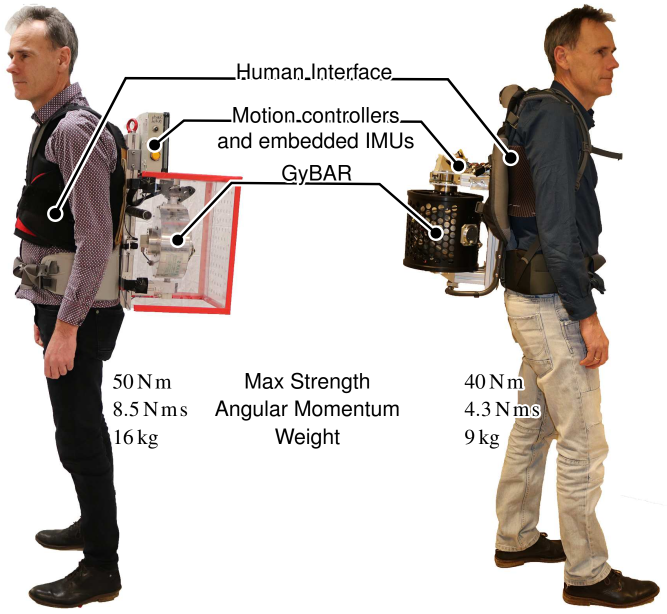

Figure S4. Model wearing different GyBAR versions. Left, First prototype version used in the experiments described in this paper. Right, Second upgraded prototype version.

To reduce abrupt perturbations occurring at saturation, safe 'singularity-robust' controllers for gyroscopic actuators have been proposed for both this specific application[16] and other generic uses and configurations[2], which improve predictability by smoothly arresting gimbal motions near geometric constraints. A simplified version of this was implemented in the GyBAR, but it is conceded that the influences of such perturbations were underestimated during selection of the parameters.

S4 Undesired balance artifacts

The prototype GyBAR was constructed to explore the principles of design and control of gyroscopic actuation, hence its mass was not optimized for wearability. This considerable weight (16 kg) was found to have several unintended consequences.

In addition to the aforementioned threat of physiological strain during prolonged load carriage, a heavy load is counterproductive to the aim of improving balance[19,20]. This is illustrated in Experiment 1, where two of the three assistive controllers were found to not be statistically better than simply removing the device. From this standpoint, it is of great interest to test a lighter, optimized GyBAR that (i) does not risk potential strain or overexertion with prolonged use and (ii) does not negate a

large portion of its potential benefits by its weight alone.

Whereas the healthy participants of this study were capable of bearing the full weight of the prototype GyBAR, the members of the chronic stroke group in Experiment 2, representing one of the primary target groups of this technology, were not. The safety system used in Experiment 2 served to mitigate the physiological strain experienced the individuals with chronic stroke.

However, the additional unloading provided by the safety system imparted stabilizing forces, potentially improving proprioception. It was expected that this unloading would not have a large impact due to the facts that (i) the height of the fixation point (approx. 2.5 m) was large in comparison to the expected horizontal deflections of the trunk, implying that the unloading force would remain primarily vertical and not directly influence horizontal CoM control, and (ii) the spring was relatively soft (460 N/m) to ensure minimal change in the magnitude of the unloading force with displacement of the trunk. However, the passive gantry-based safety system exhibited some direction dependence; it could translate easily along a rail in the AP direction, but was essentially fixed in the ML direction due to high inertia and friction, creating the possibility for subjects to exchange lateral forces with it. In the standing task with a reduced AP BoS, any such assistance from the safety system only offset the detriment that the remaining 7.5 kg weight may have had, and no significant difference in task performance was observed between conditions FR and IN (p=0.89, both groups). In the standing and walking tasks with a reduced ML BoS, however, the horizontal forces did significantly improve task performance (compare, e.g., condition FR and IN in Supplementary Fig. S7b, p=0.043). From the peak trunk angular deflections, it is estimated that the safety system may have imparted moments in the ML direction on the same order of magnitude as the GyBAR. We presume that this allowed subjects to rely less on the gyroscopic assistance, which explains why no significant differences were observed between conditions IN and D in the ML tasks of Experiment 2 ($p \geq 0.465$), but were observed in Experiment 1 for a comparable task (p=0.012).

S5 Supplementary results

S5.1 Experiment 1

Both raw and normalized primary outcome measures are shown in Supplementary Fig. S5 with all statistically significant pairs denoted. Secondary outcome measures including centroidal frequency of the trunk angle, gyroscopic moment, and gyroscopic angular impulse are shown in Supplementary Fig. S6. Additional outcome measures are shown in Supplementary Table SIV.

The presence of temporal effects was evaluated by means of comparing the task performance in a baseline condition at the beginning and end of the experiment (Supplementary Table SV).

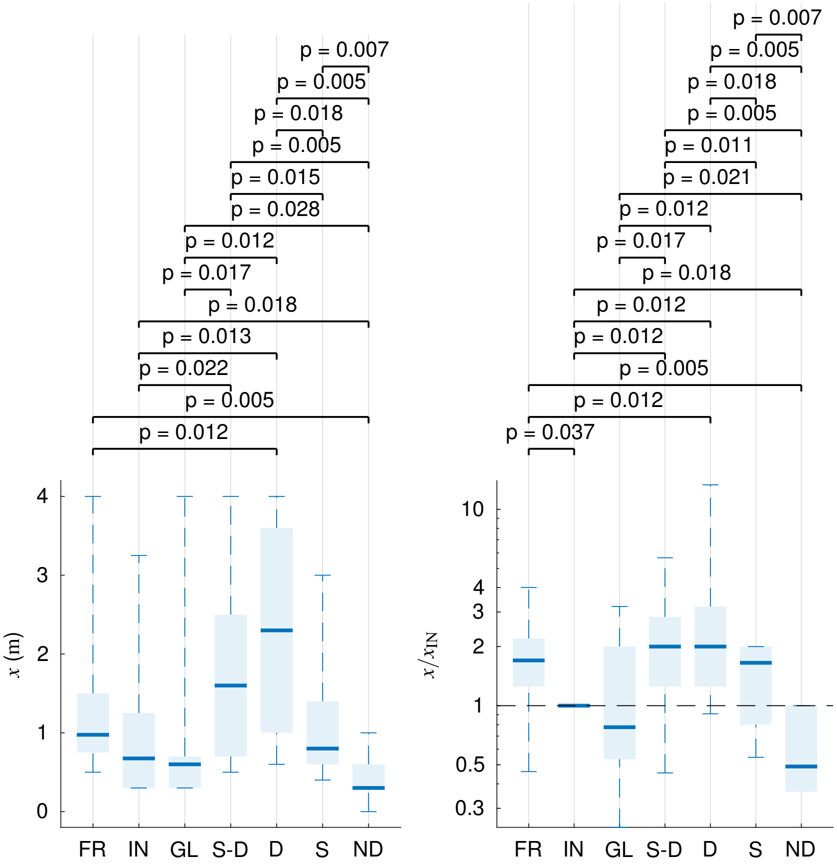

(a) **(b)**

Figure S5. Experiment 1 primary outcome measures. Distance walked along a beam of width 3 cm and length 4 m, showing all statistically significant pairs ($p < 0.05$) for conditions 'free' (FR), 'inactive' (IN), 'gimbal locked' (GL), 'spring-damper' (S-D), 'damper' (D), 'spring' (S), and 'negative damper' (ND). (a) original units. (b) normalized with respect to condition 'inactive' (displayed in a logarithmic scale).

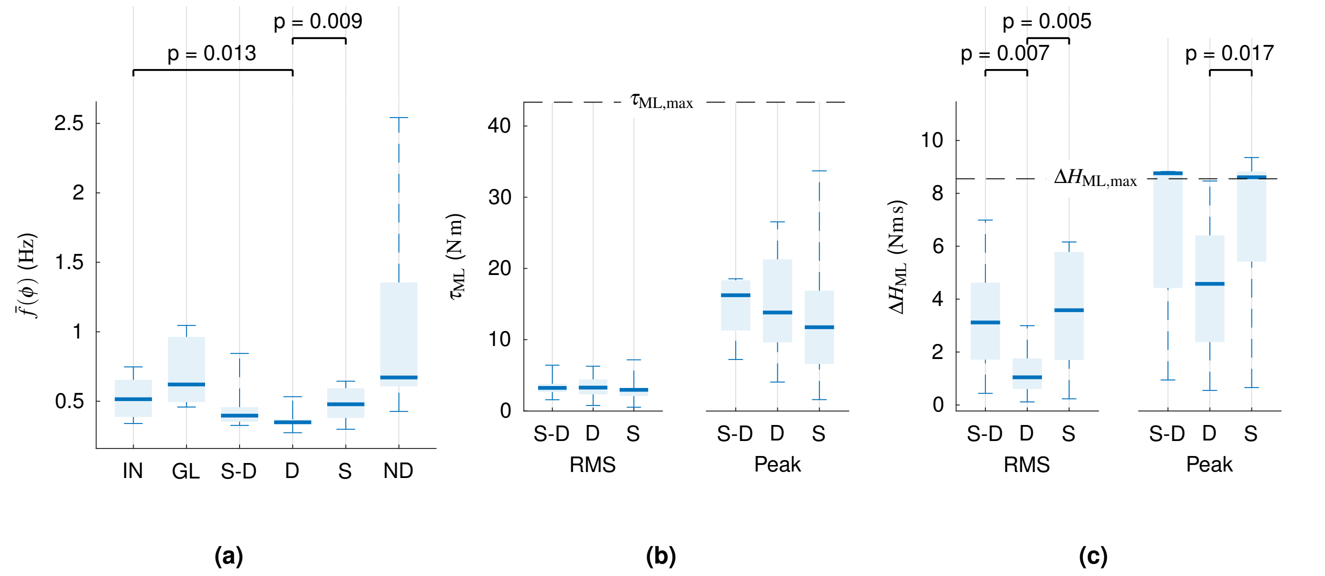

Figure S6. Experiment 1 secondary outcome measures for conditions 'free' (FR), 'inactive' (IN), 'gimbal locked' (GL), 'spring-damper' (S-D), 'damper' (D), 'spring' (S), and 'negative damper' (ND). Statistical significance between conditions ($p < 0.05$) is shown with a top bracket. (a) centroidal frequency of the trunk roll angle. (b) RMS and peak gyroscopic moment. (c) RMS and peak exchanged angular momentum.

Table SIV. Experiment 1 group metrics.

Outcome measures	Symbol	Units	$\tilde{x}(^{P_{75}}_{P_{25}})$ [1] 'free' (FR)	'inactive' (IN)	'gimbal locked' (GL)	'spring-damper' (S-D)	'damper' (D)	'spring' (S)	'negative damper' (ND)
**Distance walked**, x									
Raw	x	m	$0.975(^{1.500}_{0.750})$	$0.675(^{1.250}_{0.300})$	$0.600(^{0.700}_{0.300})$	$1.600(^{2.500}_{0.700})$	$2.300(^{3.600}_{1.000})$	$0.800(^{1.400}_{0.600})$	$0.300(^{0.600}_{0.300})$
Normalized [2]	x/x_{IN}	-	$1.7(^{2.2}_{1.25})$	1	$0.777(^{2}_{0.533})$	$2(^{2.83}_{1.25})$	$2(^{3.2}_{1.25})$	$1.65(^{2}_{0.8})$	$0.49(^{1}_{0.364})$
**Trunk roll angle**, ϕ									
RMS	ϕ_{RMS}	deg	-	$3.499(^{6.655}_{2.792})$	$3.563(^{4.838}_{2.376})$	$3.636(^{4.439}_{2.164})$	$5.613(^{6.421}_{3.924})$	$3.650(^{9.982}_{2.763})$	$3.478(^{4.527}_{1.492})$
Peak	ϕ_{peak}	deg	-	$7.933(^{15.916}_{5.646})$	$9.674(^{11.997}_{4.807})$	$9.285(^{18.738}_{5.984})$	$13.083(^{15.409}_{12.335})$	$11.495(^{22.993}_{6.726})$	$7.654(^{14.297}_{3.165})$
Excursion	$\Delta(\phi)$	deg	-	$10.562(^{28.449}_{7.628})$	$12.646(^{18.055}_{4.586})$	$12.108(^{25.461}_{9.414})$	$22.073(^{24.184}_{15.945})$	$18.220(^{30.779}_{10.520})$	$9.351(^{18.001}_{3.017})$
Centroidal Frequency	$\bar{f}(\phi)$	Hz	-	$0.515(^{0.654}_{0.387})$	$0.621(^{0.963}_{0.494})$	$0.397(^{0.458}_{0.352})$	$0.349(^{0.370}_{0.343})$	$0.478(^{0.594}_{0.380})$	$0.671(^{1.355}_{0.607})$
**Trunk roll angular velocity**, $\dot{\phi}$									
RMS	$\dot{\phi}_{\mathrm{RMS}}$	deg/s	-	$5.519(^{10.554}_{3.848})$	$8.370(^{10.414}_{7.393})$	$6.008(^{7.893}_{4.774})$	$8.892(^{10.699}_{7.339})$	$10.220(^{13.830}_{7.182})$	$7.529(^{14.149}_{4.953})$
Peak	$\dot{\phi}_{\mathrm{peak}}$	deg/s	-	$17.398(^{47.522}_{14.357})$	$28.691(^{35.520}_{16.726})$	$24.357(^{32.730}_{17.240})$	$28.972(^{34.741}_{21.406})$	$36.099(^{65.669}_{20.872})$	$23.460(^{51.215}_{14.788})$
**Gyroscopic moment**, τ_{ML}									
RMS	τ_{RMS}	Nm	-	-	$0.136(^{0.201}_{0.019})$	$3.219(^{3.797}_{2.645})$	$3.263(^{4.413}_{2.307})$	$2.947(^{3.026}_{2.065})$	$1.456(^{2.439}_{0.756})$
Percentage of Max		%	-	-	$0.314(^{0.463}_{0.0435})$	$7.43(^{8.76}_{6.1})$	$7.53(^{10.2}_{5.32})$	$6.8(^{6.98}_{4.76})$	$3.36(^{5.63}_{1.74})$
Peak	τ_{peak}	Nm	-	-	$0.993(^{1.196}_{0.177})$	$16.234(^{18.337}_{11.288})$	$13.809(^{21.282}_{9.616})$	$11.740(^{16.864}_{6.590})$	$7.341(^{9.341}_{2.740})$
Percentage of Max		%	-	-	$2.29(^{2.76}_{0.408})$	$37.5(^{42.3}_{26})$	$31.9(^{49.1}_{22.2})$	$27.1(^{38.9}_{15.2})$	$16.9(^{21.6}_{6.32})$
**Exchanged angular momentum**, ΔH_{ML}									
RMS	ΔH_{RMS}	Nms	-	-	$0.032(^{0.039}_{0.019})$	$3.119(^{4.620}_{1.712})$	$1.046(^{1.761}_{0.605})$	$3.583(^{5.785}_{1.702})$	$0.284(^{0.819}_{0.126})$
Percentage of Max		%	-	-	$0.337(^{0.478}_{0.0243})$	$34.7(^{54.1}_{18.9})$	$12.2(^{20.7}_{7.07})$	$41.5(^{64.3}_{20.5})$	$3.34(^{9.39}_{1.69})$
Peak	ΔH_{peak}	Nms	-	-	$0.106(^{0.135}_{0.022})$	$8.760(^{8.819}_{4.425})$	$4.579(^{6.410}_{2.374})$	$8.609(^{8.828}_{5.421})$	$1.098(^{2.717}_{0.384})$
Percentage of Max		%	-	-	$1.1(^{1.61}_{0.0546})$	$102(^{102}_{44.2})$	$53.4(^{75.3}_{27.6})$	$98.1(^{99.5}_{64.1})$	$11.3(^{30.9}_{4.7})$
**Geometric Saturation**									
Ocurrences [3]		% (n)	-	-	-	70 (7)	10 (1)	60 (6)	0 (0)
Lead to failure		% (n)	-	-	-	71.4 (5)	0 (0)	83.3 (5)	0 (0)
Exchanged mean power	$\bar{P}$	W	-	-	$-0.011(^{-0.001}_{-0.020})$	$-0.249(^{-0.163}_{-0.319})$	$-0.416(^{-0.225}_{-0.734})$	$-0.152(^{-0.107}_{-0.229})$	$0.164(^{0.462}_{0.057})$
Perception AHP ranking [4]			-	-	3	2	1	4	5

[1] Median of the recorded data with its 25th and 75th percentiles showed in parentheses.
[2] With respect to baseline condition 'inactive' (IN).
[3] Percentage of subjects who experienced geometric saturation (number of subjects in parentheses).
[4] Ranking from best (1) to worst (5) using the Analytic Hierarchy Process (AHP).

Table SV. Experiment 1 learning effects.

Condition	Trial	$\tilde{x}(^{P_{75}}_{P_{25}})$ Distance walked (m)	Learning Rate [1]
'free' (FR)	Initial	$0.775(^{1.600}_{0.600})$	$1.267(^{1.667}_{1.000})$
	Final	$1.050(^{2.100}_{0.750})$	
'inactive' (IN)	Initial	$0.600(^{1.250}_{0.300})$	$0.860(^{1.833}_{0.500})$
	Final	$0.675(^{1.000}_{0.300})$	

[1] Computed as the median of 'Final'/'Initial' for each subject. Values > 1 indicate possible learning.

S5.2 Experiment 2

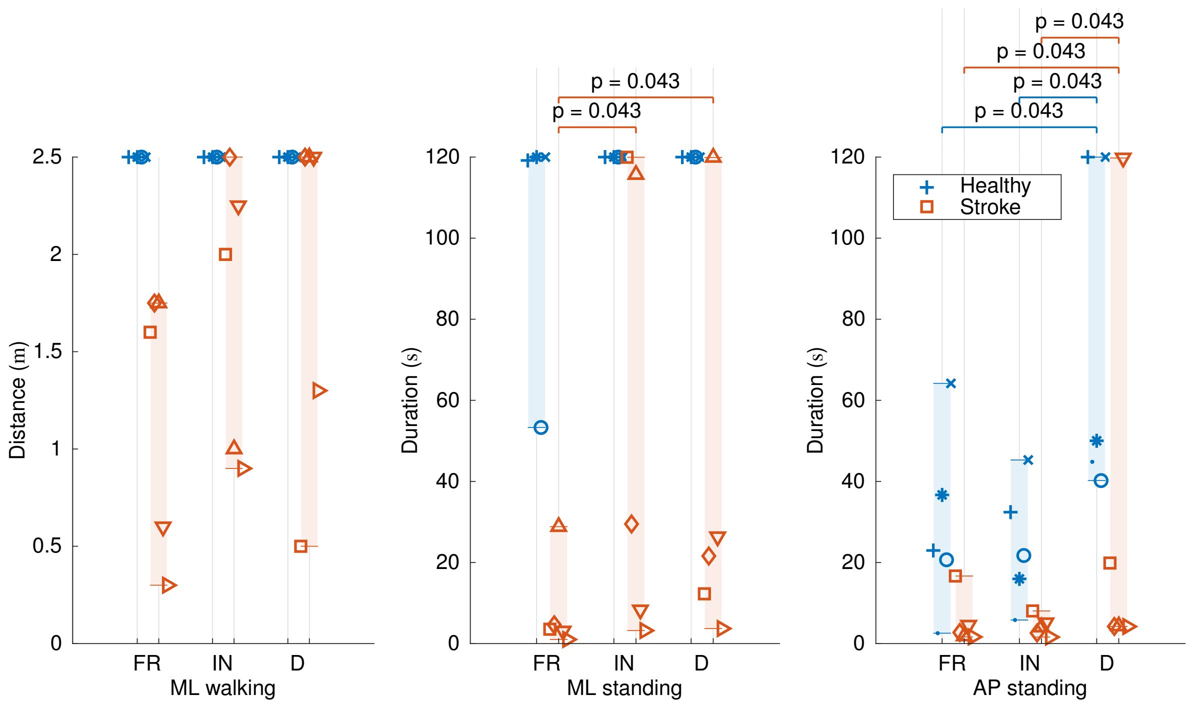

(a) **(b)** **(c)**

Figure S7. Experiment 2 primary outcome measures for most difficult level of balancing task completed per subject. Both stroke survivors (pairwise left, blue) and healthy controls (pairwise right, red) are shown, for conditions 'free' (FR), 'inactive' (IN), and 'damper' (D). Within-group significant differences ($p < 0.05$) are denoted by brackets. Note that, in contrast to Experiment 1, part of the weight of the GyBAR was unloaded in all conditions in which it was worn (IN and D) to avoid overburdening the stroke subjects. In each plot, the upper boundary represents the maximum attainable score for that task. The ML task results were not analyzed due to prevalent ceiling effects and significant improvement between conditions FR and IN, indicating that the unloading system was exploited for stabilization.

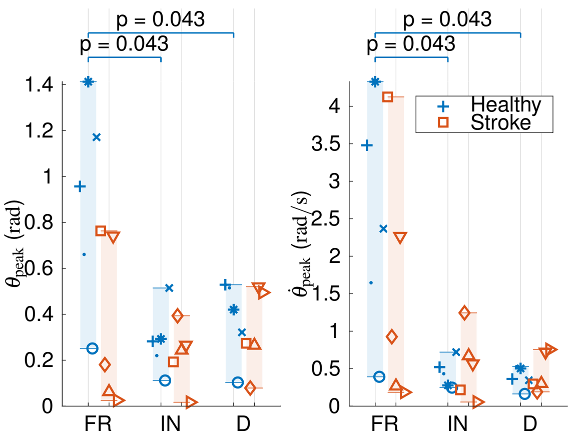

(a)

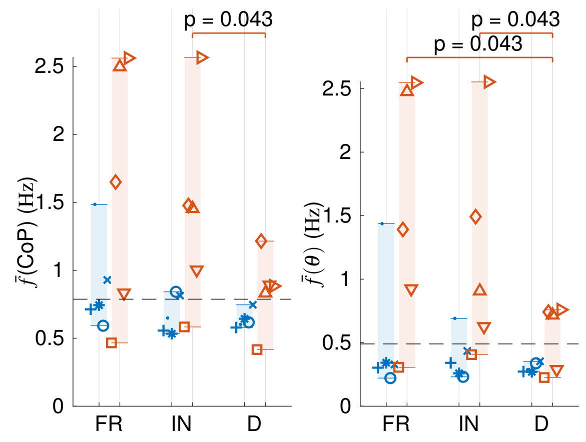

(b)

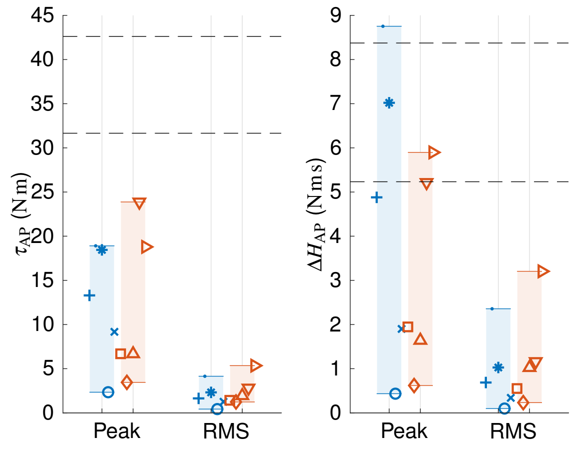

(c)

Figure S8. Experiment 2 secondary outcome measures for the AP balancing task. (a) Peak trunk pitch angle (θ) and angular velocity. Significant differences in the healthy population between 'free' (FR) and 'inactive' (IN) may be due to the added mass/inertia of the GyBAR or exploitation of the accompanying unloading system (note, however, that such differences are not present in the primary outcome measures). (b) Centre of pressure (CoP) and trunk pitch angle (θ) centroidal sway frequencies. Median healthy full-BoS frequencies for the IN condition are denoted by dotted lines. (c) Gyroscopic moment τ and exchanged angular momentum ΔH. The dashed lines correspond to the angular momentum limits of the asymmetric gimbal stops. Subjects H2 (•), H3 (✱), and S5 (▷) encountered geometric saturation and subsequent loss of assistance.

Table SVI. Experiment 2 group metrics (AP standing task only).

Metric	Symbol	Units	$\tilde{x}\binom{P_{75}}{P_{25}}$ [1]					
			Healthy			Stroke		
			'free' (FR)	'inactive' (IN)	'damper' (D)	'free' (FR)	'inactive' (IN)	'damper' (D)
**Duration**								
Raw	t	s	$22.964\binom{43.546}{16.141}$	$21.727\binom{35.637}{13.420}$	$50.024\binom{120.000}{43.674}$	$2.735\binom{7.581}{1.810}$	$4.061\binom{5.869}{2.346}$	$4.334\binom{44.843}{4.216}$
Normalized[2]	t/t_{IN}	-	$0.951\binom{1.637}{0.641}$	$1.000\binom{1.000}{1.000}$	$3.135\binom{4.705}{2.450}$	$1.025\binom{1.308}{0.779}$	$1.000\binom{1.000}{1.000}$	$2.466\binom{7.798}{1.487}$
**Trunk pitch angle**, θ								
RMS	θ_{RMS}	rad	$0.195\binom{0.386}{0.158}$	$0.084\binom{0.096}{0.066}$	$0.069\binom{0.147}{0.065}$	$0.182\binom{0.226}{0.077}$	$0.075\binom{0.141}{0.055}$	$0.214\binom{0.242}{0.122}$
Peak	θ_{peak}	rad	$0.738\binom{0.861}{0.631}$	$0.194\binom{0.233}{0.149}$	$0.265\binom{0.288}{0.174}$	$0.352\binom{0.500}{0.142}$	$0.158\binom{0.262}{0.090}$	$0.338\binom{0.353}{0.259}$
RMS vel	$\dot{\theta}_{\mathrm{RMS}}$	rad/s	$0.292\binom{0.524}{0.237}$	$0.129\binom{0.186}{0.078}$	$0.085\binom{0.121}{0.068}$	$0.240\binom{0.486}{0.093}$	$0.287\binom{0.358}{0.066}$	$0.119\binom{0.169}{0.091}$
Peak vel	$\dot{\theta}_{\mathrm{peak}}$	rad/s	$2.367\binom{3.692}{1.333}$	$0.433\binom{0.571}{0.270}$	$0.363\binom{0.509}{0.300}$	$0.926\binom{2.730}{0.247}$	$0.566\binom{0.814}{0.177}$	$0.301\binom{0.731}{0.270}$
Centroidal frequency	$\bar{f}(\theta)$	Hz	$0.329\binom{0.616}{0.283}$	$0.341\binom{0.499}{0.252}$	$0.288\binom{0.342}{0.272}$	$1.391\binom{2.493}{0.770}$	$0.906\binom{1.757}{0.572}$	$0.717\binom{0.746}{0.273}$
**Centre of pressure**, CoP								
RMS	$\mathrm{CoP}_{\mathrm{RMS}}$	mm	$7.194\binom{8.140}{6.396}$	$6.758\binom{7.214}{4.724}$	$5.867\binom{6.804}{4.461}$	$7.718\binom{13.299}{3.758}$	$6.305\binom{8.045}{3.309}$	$5.669\binom{11.299}{4.876}$
Peak	$\mathrm{CoP}_{\mathrm{peak}}$	mm	$17.077\binom{20.814}{13.394}$	$14.100\binom{17.288}{11.778}$	$15.950\binom{19.205}{12.008}$	$13.667\binom{24.617}{8.347}$	$15.828\binom{18.351}{7.822}$	$16.211\binom{23.487}{9.340}$
RMS vel	$\frac{\mathrm{d}}{\mathrm{d}t}\mathrm{CoP}_{\mathrm{RMS}}$	mm/s	$54.077\binom{60.904}{49.637}$	$50.609\binom{54.296}{36.142}$	$39.837\binom{44.032}{37.024}$	$51.004\binom{113.281}{49.753}$	$76.940\binom{80.634}{34.782}$	$51.867\binom{64.415}{44.326}$
Peak vel	$\frac{\mathrm{d}}{\mathrm{d}t}\mathrm{CoP}_{\mathrm{peak}}$	mm/s	$215.225\binom{252.978}{182.600}$	$167.883\binom{212.468}{139.032}$	$172.109\binom{211.995}{143.182}$	$214.822\binom{302.070}{174.384}$	$220.309\binom{253.768}{120.204}$	$199.492\binom{326.645}{168.328}$
Centroidal frequency	$\bar{f}(\mathrm{CoP})$	Hz	$0.814\binom{1.101}{0.746}$	$0.689\binom{0.790}{0.609}$	$0.768\binom{0.803}{0.597}$	$1.696\binom{4.063}{0.716}$	$1.475\binom{2.071}{0.929}$	$0.878\binom{1.008}{0.718}$
**Gyroscopic moment**, τ_{AP}								
RMS	τ_{RMS}	Nm	-	-	$1.636\binom{2.774}{1.049}$	-	-	$1.934\binom{3.445}{1.373}$
Peak	τ_{peak}	Nm	-	-	$13.296\binom{18.565}{7.463}$	-	-	$6.687\binom{20.061}{5.870}$
**Exchanged angular momentum**, ΔH_{AP}								
RMS	ΔH_{RMS}	Nms	-	-	$0.687\binom{1.359}{0.279}$	-	-	$1.024\binom{1.672}{0.471}$
Peak	ΔH_{peak}	Nms	-	-	$4.879\binom{7.453}{1.533}$	-	-	$1.946\binom{5.387}{1.385}$

[1] Median value with 25th and 75th percentiles shown in parentheses. For vector quantities, only the AP component is reported.

[2] With respect to baseline condition 'inactive' (IN).

Note that in Experiment 2 the weight of the GyBAR was partially unloaded in conditions 'inactive' (IN) and 'damper' (D) for both groups, whereas in Experiment 1 the full weight was borne in all conditions but 'free' (FR).

Table SVII. Experiment 2 investigation of temporal effects for the AP standing task.

Subject		Group[1]	t_D/t_{IN}	$\dot{\theta}_{peak,D}/\dot{\theta}_{peak,IN}$	RPE_D-RPE_{IN}[2]
H1	+	A	3.70	0.70	+0
H2	•	A	7.71	1.22	+1
H3	∗	A	3.14	1.81	-1
H4	○	B	1.85	0.66	+0
H5	×	B	2.65	0.48	+1
S1	□	B	2.47	1.36	+2
S2	◇	A	1.63	0.15	+0
S3	△	A	1.07	0.45	+2
S4	▽	B	23.30	1.28	+0
S5	▷	B	2.63	13.61	-1

[1] Group testing orders. Group A: FR-IN-D. Group B: FR-D-IN.
[2] An increase in Borg rating (RPE) indicates greater perceived exertion[21].

References

1. Lemus, D., van Frankenhuyzen, J. & Vallery, H. Design and Evaluation of a Balance Assistance Control Moment Gyroscope. *J. Mech. Robotics* **9**, 051007–051007–9, DOI: 10.1115/1.4037255 (2017).
2. Valk, L., Berry, A. & Vallery, H. Directional Singularity Escape and Avoidance for Single-Gimbal Control Moment Gyroscopes. *J. Guid. Control. Dyn.* **0**, 1–14, DOI: 10.2514/1.G003132 (2018).
3. Sawers, A. & Ting, L. H. Beam walking can detect differences in walking balance proficiency across a range of sensorimotor abilities. *Gait & Posture* **41**, 619–623, DOI: 10.1016/j.gaitpost.2015.01.007 (2015).
4. Nøkland, H. *Nonlinear Observer Design for GNSS and IMU Integration*. MSc thesis, Norwegian University of Science and Technology, Trondheim, Norway (2011).
5. Berg, K., Wood-Dauphine, S., Williams, J. I. & Gayton, D. Measuring balance in the elderly: Preliminary development of an instrument. *Physiother. Can.* DOI: 10.3138/ptc.41.6.304 (2009).
6. Wrisley, D. M., Marchetti, G. F., Kuharsky, D. K. & Whitney, S. L. Reliability, Internal Consistency, and Validity of Data Obtained With the Functional Gait Assessment. *Phys. Ther.* **84**, 906–918, DOI: 10.1093/ptj/84.10.906 (2004).
7. Cohen, H., Blatchly, C. A. & Gombash, L. L. A Study of the Clinical Test of Sensory Interaction and Balance. *Phys. Ther.* **73**, 346–351, DOI: 10.1093/ptj/73.6.346 (1993).
8. Romberg, M. H. *A Manual of the Nervous Diseases of Man*, vol. 2 (Sydenham Society, 1853).
9. Horak, F. B. & Nashner, L. M. Central programming of postural movements: Adaptation to altered support-surface configurations. *J. Neurophysiol.* **55**, 1369–1381, DOI: 10.1152/jn.1986.55.6.1369 (1986).
10. Bruininks, R. *Bruininks-Oseretsky Test of Motor Proficiency: Examiner's Manual* (American Guidance Service, 1978).
11. Villamonte, R. *et al.* Reliability of 16 Balance Tests in Individuals with down Syndrome. *Percept. Mot. Ski.* **111**, 530–542, DOI: 10.2466/03.10.15.25.PMS.111.5.530-542 (2010).
12. Sawers, A. & Hafner, B. J. A study to assess whether fixed-width beam walking provides sufficient challenge to assess balance ability across lower limb prosthesis users. *Clin. Rehabil.* **32**, 483–492, DOI: 10.1177/0269215517732375 (2018).
13. Al-Khabbaz, Y. S., Shimada, T. & Hasegawa, M. The effect of backpack heaviness on trunk-lower extremity muscle activities and trunk posture. *Gait & Posture* **28**, 297–302, DOI: 10.1016/j.gaitpost.2008.01.002 (2008).
14. Simpson, K. M., Munro, B. J. & Steele, J. R. Effect of load mass on posture, heart rate and subjective responses of recreational female hikers to prolonged load carriage. *Appl. Ergonomics* **42**, 403–410, DOI: 10.1016/j.apergo.2010.08.018 (2011).
15. Holewijn, M. Physiological strain due to load carrying. *Eur. J. Appl. Physiol. Occup. Physiol.* **61**, 237–245, DOI: 10.1007/BF00357606 (1990).

16. Berry, A., Lemus, D., Babuška, R. & Vallery, H. Directional Singularity-Robust Torque Control for Gyroscopic Actuators. *IEEE/ASME Transactions on Mechatronics* **21**, 2755–2763, DOI: 10.1109/TMECH.2016.2603601 (2016).

17. Brown, D. & Peck, M. A. Scissored-Pair Control-Moment Gyros: A Mechanical Constraint Saves Power. *J. Guid. Control. Dyn.* **31**, 1823–1826, DOI: 10.2514/1.37723 (2008).

18. Chiu, J. & Goswami, A. Design of a Wearable Scissored-Pair Control Moment Gyroscope (SP-CMG) for Human Balance Assist. In *Volume 5A: 38th Mechanisms and Robotics Conference*, V05AT08A023, DOI: 10.1115/DETC2014-35539 (American Society of Mechanical Engineers, Buffalo, New York, USA, 2014).

19. Schiffman, J. M., Bensel, C. K., Hasselquist, L., Gregorczyk, K. N. & Piscitelle, L. Effects of carried weight on random motion and traditional measures of postural sway. *Appl. Ergonomics* **37**, 607–614, DOI: 10.1016/j.apergo.2005.10.002 (2006).

20. Heller, M. F., Challis, J. H. & Sharkey, N. A. Changes in postural sway as a consequence of wearing a military backpack. *Gait & Posture* **30**, 115–117, DOI: 10.1016/j.gaitpost.2009.02.015 (2009).

21. Borg, G. A. Pyschophysical bases of physical exertion. *Medicine Sci. Sports Exerc.* **14**, 377–381 (1982).
